# Two of a Kind: Composition and Photophysics of Two Silver Nanoclusters Stabilized by the Same DNA Sequence

**DOI:** 10.1021/jacs.6c04812

**Published:** 2026-06-18

**Authors:** Letizia Liccardo, Hiroki Kanazawa, Giacomo Romolini, Cecilia Cerretani, Simon Wentzel Lind, Beatrice Polido, Christian Brinch Mollerup, Vanessa Rück, Zhiyu Huang, Leila Lo Leggio, Jiro Kondo, Tom Vosch

**Affiliations:** † Department of Chemistry, 4321 University of Copenhagen, Universitetsparken 5, 2100 Copenhagen, Denmark; ‡ Department of Materials and Life Sciences, 13070 Sophia University, 7-1 Kioi-cho, Chiyoda-ku, 102-8554 Tokyo, Japan; § Department of Molecular Sciences and Nanosystems, Ca’ Foscari University of Venice, Via Torino 155, 30170 Mestre, Venezia, Italy; ∥ Department of Forensic Medicine, 4321 University of Copenhagen, Frederik V’s Vej 11, 2100 Copenhagen, Denmark

## Abstract

Sequence–structure relationships in DNA-stabilized silver nanoclusters (DNA-AgNCs) remain a central challenge in predicting their optical properties. Here, we show that a single guanine-rich DNA scaffold can direct the formation of two distinct silver nanoclusters within the same reaction mixture. While the clusters’ composition and charge are very similar, their photophysical properties are surprisingly different. We present, for the first time, detailed structural insights into one of the two DNA-AgNCs: the near-infrared emitting silver nanocluster with a predominantly microsecond-lived excited state, DNA_2_[Ag_17+2_]^13+^. Remarkably, two guanines bridge the central 17-silver-atom cluster with two additional isolated silver atoms, which are further stabilized through interactions with cytosine and adenine nucleobases. These findings provide new insight into how DNA sequences drive AgNC formation and tune spectroscopic properties, advancing the rational design of near-infrared-emissive nanoclusters with tailored optical responses.

## Graphical abstract

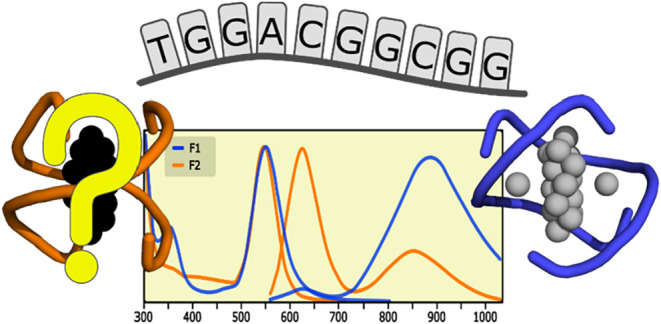

## Introduction

DNA-stabilized silver nanoclusters (DNA-AgNCs) represent a unique class of hybrid nanomaterials with great potential in bioimaging and biosensing applications.^1–6^ Typically, one to three DNA oligomers (10 to 16 nucleobases each) coordinate 10–30 silver atoms and cations, effectively encoding the AgNC structure and photophysical properties.^7–14^ A single DNA sequence can offer many potential binding sites that allow for the formation and stabilization of AgNCs with a range of stoichiometries.^15–17^ As a result, the synthesis of DNA-AgNCs usually yields a complex reaction mixture comprising multiple DNA-AgNCs along with several byproducts, making high-performance liquid chromatography (HPLC) essential for isolating the species of interest.^18^ Despite this inherent complexity and the range of compounds formed in the as-synthesized solution, a single HPLC-stable DNA-AgNC is typically collected and analyzed.^18^ For the first time, we isolated and compared two DNA-AgNCs that are compositionally related yet display different luminescence behaviors. This observation indicates that their optical response is governed not only by the DNA sequence and the number of silver atoms and cations, but also by the distinct conformational imprint imposed by the DNA scaffold.

Both DNA-AgNCs are stabilized by the same two DNA strands (5′-TGG ACG GCG G-3′) that embed 6 neutral silver atoms along with 12 to 13 Ag^+^ cations. One of the clusters was previously reported by Rück et al.,^19,20^ exhibiting excitation-intensity-dependent dual emission in the red and near-infrared (NIR) ranges, whereas the newly identified species mainly displays μs-lived luminescence centered around 880 nm. These two DNA-AgNCs coexist in the same as-synthesized solution and can be separated chromatographically due to their distinct retention times. Because their hydrodynamic volumes could not be determined from time-resolved anisotropy measurements, compositional insights relied primarily on mass spectrometry and, for one of the DNA-AgNCs, the structure was determined by single-crystal X-ray diffraction measurements. Both species crystallized, but the crystals of the dual-emissive DNA-AgNC proved highly unstable, degrading rapidly under illumination during microscope inspection. In contrast, the crystals of the new, mainly μs-lived luminescent DNA-AgNC displayed good light stability, allowing successful structural characterization that revealed novel interactions. Here, we provide the first structural evidence of coordinate bond formation between silver atoms and thymine nucleobases within DNA-AgNCs at pH 7. So far, such interaction was only suggested to occur at more basic pH.^21^

## Results and Discussion

### Synthesis and HPLC Purification

Both DNA-AgNCs were formed in the same solution by mixing 30 μM DNA, 150 μM AgNO_3_ and 75 μM NaBH_4_ in 10 mM ammonium acetate (NH_4_OAc). After 6 days at 4 °C, the solution was purified by reversed-phase HPLC, and the two DNA-AgNCs were collected separately. Figure 1 shows the chromatograms of the mixture. Fraction 1 (F1) displays emission exclusively at 830 nm, whereas Fraction 2 (F2)—previously investigated by Rück et al.—exhibits emissions at both 630 and 830 nm.

**Figure 1:**
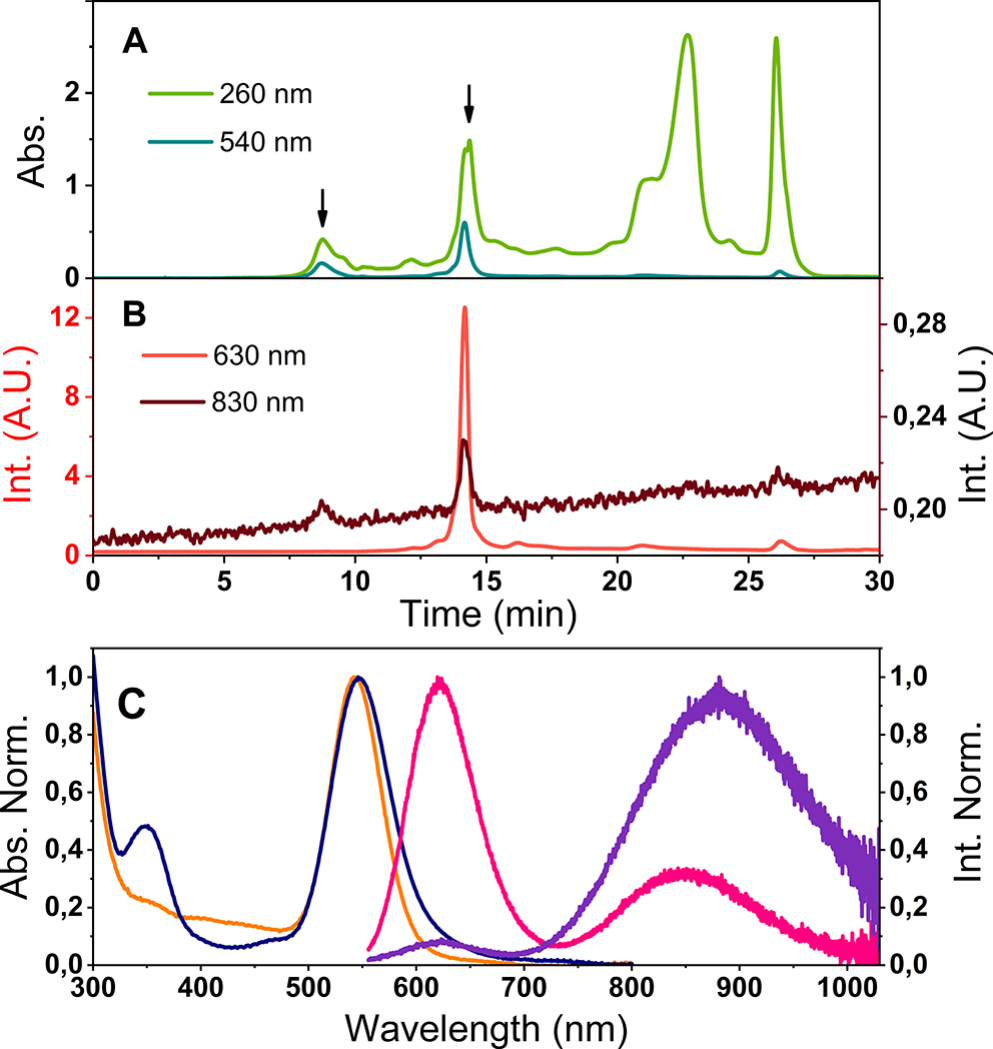
(A) and (B) HPLC chromatograms of the solution with F1 and F2. (A) HPLC chromatograms monitoring the absorbance at 260 nm (green) and 540 nm (blue). (B) Chromatograms monitoring the emission at 630 nm (light red) and 830 nm (dark red) upon excitation at 540 nm. (C) Normalized absorption and emission spectra of F1 (blue and purple, respectively) and F2 (orange and pink, respectively) in 10 mM NH_4_OAc at room temperature. The emission was acquired exciting at 514 nm.

### Photophysical Properties

F1 and F2 share a similar but slightly shifted main absorption peak around 545 nm (Figure 1C). Compared to F2, F1 exhibits a wider full width at half-maximum (FWHM) for this absorption feature, as well as an additional absorption peak at ∼350 nm (see Table 1 and Figure 1C). As reported previously, F2 displays dual emission, with a subnanosecond-lived fluorescence band at 623 nm and a μs-lived luminescence band at 850 nm. As expected, excitation spectra recorded at 620 and 800 nm (Figure 2B) overlay and match the absorption spectrum, confirming that both bands originate from the same DNA-AgNC.^19^ On the other hand, F1 shows primarily μs-lived luminescence at 881 nm, with a very weak fluorescence band at 623 nm. As shown in Figure 2A, the excitation spectra recorded at 620 and 800 nm do not match. Specifically, the excitation spectrum at 800 nm closely resembles the absorption spectrum of F1, whereas the excitation spectrum at 620 nm aligns more with the absorption spectrum of F2. These results suggest that a minor contamination of the F2 emitter is present in the F1 sample and is responsible for most of the emission observed at 620 nm (see Figure S4 for more details).

**Figure 2:**
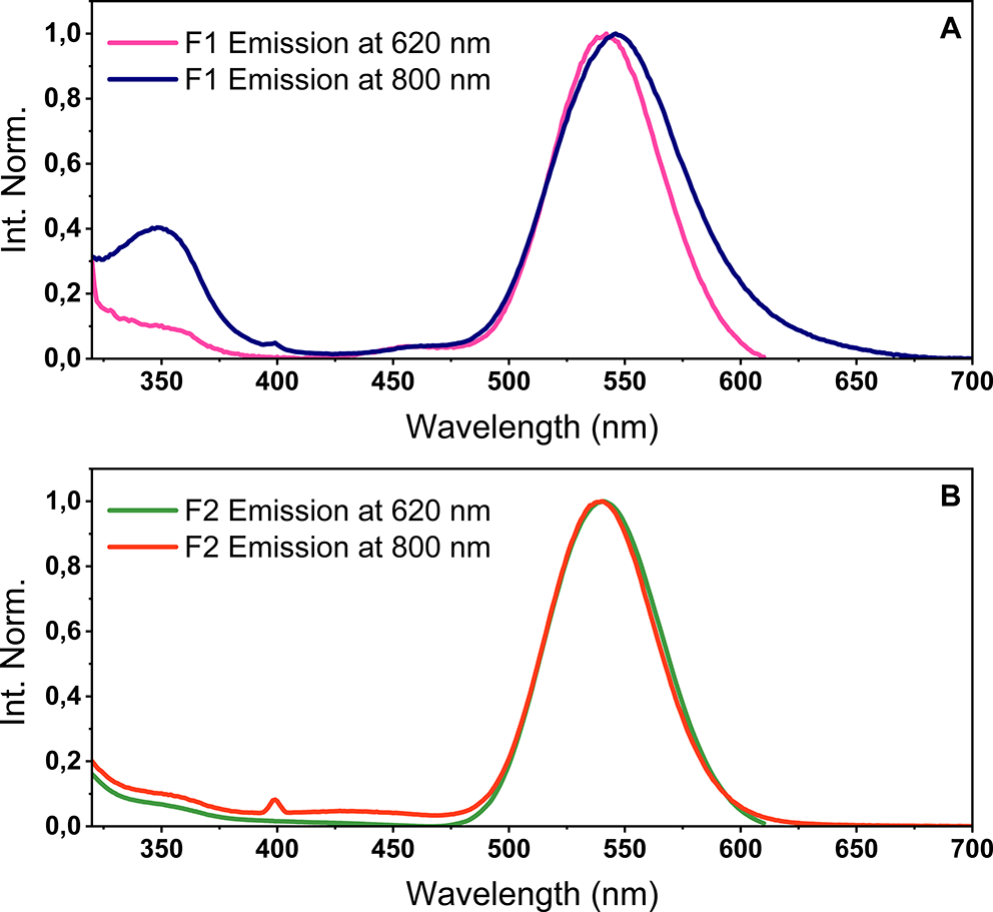
(A) Excitation spectra of F1 in 10 mM NH_4_OAc at room temperature, monitoring the emission at 620 nm (pink) and 800 nm (dark blue). (B) Excitation spectra of F2 in 10 mM NH_4_OAc at room temperature, monitoring the emission at 620 nm (green) and 800 nm (orange). The small peak at 400 nm is an artifact from monitoring the emission at 800 nm.

**Table 1:** Steady-State Absorption and Emission Properties of F1 and F2

sample	λ_abs_ (nm)	FWHM_abs_ (nm)	FWHM_exc_ (nm)		λ_fl_ (nm)	λ_lu_ (nm)	ϕ
F1	547	67	55^b^	66^c^	623	881	0,016
F2	543	58	57^b^	55^c^	623	850	0,02[Table-fn t1fn4]

λ_abs_, λ_fl_, and λ_lu_ indicate the absorption, fluorescence and luminescence maxima, respectively. ϕ represents the quantum yield of the main emission peak, i.e., the ∼880 nm luminescence band for F1 and the ∼620 nm fluorescence band for F2. FWHM_abs_ of the absorption peak. FWHM_exc_ of the excitation peak monitoring at

a 620 nm and

b 800 nm. Emission spectra were measured exciting at 530 nm.

c Data taken from Rück et al.^19^

In Figures 1C and 2A, it is also evident that the F1 sample contains an additional small impurity with an absorption feature around 460 nm (See Figure S1 for the emission spectrum, excited at 460 nm). Although both F1 and F2 are stable for several months when stored at 4 °C in the dark, they exhibit limited chemical stability at 25 °C if not carefully protected from light exposure, solvent evaporation, and atmospheric exchange. As shown in Figures S10 and S11 panels A and B, without strict light protection and not sealed with parafilm, both F1 and F2 decompose over a period of 1 week, leading to the appearance of similar new absorption features between 400 and 500 nm. In contrast, sealing the cuvettes with parafilm and maintaining the samples in the dark significantly improve their stability over time (Figure S10 and S11 panels C and D).

Emission quantum yields of the main emission bands are presented in Table 1 and are 0,016 and 0,02 for F1 and F2, respectively (see Figures S2 and S3 for details). Moreover, by subtracting the rescaled F2 emission spectrum from the F1 emission spectrum, we estimated the F2 impurity in the F1 sample to be approximately 3,5% (see Supporting Information for details).

Moving from steady-state to time-resolved properties, the μs-lived decay time of F1, monitored at 800 nm, was found to be 22 μs, which is about three times shorter than that of F2 (67 μs). The fluorescence decay time at around 620 nm was found to be instrument response function (IRF)-limited for both F1 and F2. However, a lifetime of 90 ps for F2 in 10 mM NH_4_OAc aqueous solution was previously determined using femtosecond transient absorption measurements.^19^ In water, nonradiative decay pathways can occur via energy transfer from the excited state to vibrational overtones and combination bands of O–H stretching modes that overlap with the NIR emission range of the clusters. Replacing H_2_O with D_2_O shifts these vibrational modes to lower frequencies (i.e., longer wavelengths), thereby reducing vibrational quenching.^22^ In line with this concept, after solvent exchange to 10 mM NH_4_OAc in D_2_O, the fluorescence lifetimes remained too short to be resolved by our time-correlated single-photon counting (TCSPC) instrument, whereas both DNA-AgNCs exhibit longer luminescence decay times (Table 2). Upon cooling to −196 °C, however, nonradiative decay pathways were suppressed in both fractions, increasing the fluorescence lifetime to a few nanoseconds and the luminescence lifetime to hundreds of microseconds (see Table 2 for exact values). For F2, the most remarkable spectroscopic feature is the pronounced decrease in the ratio between the maximum intensity of the μs-lived band and the ns-lived band (*I*_lu_/*I*_fl_ from 0,24 to 0,11 in H_2_O and 0,76 to 0,08 in D_2_O, see Figure S9), along with a blue-shift upon cooling the solutions to −196 °C (see Figure S9). The change in *I*_lu_/*I*_fl_ ratio could suggest the presence of an energy barrier separating the initially populated excited state (Franck–Condon or fluorescent state) from the emissive state responsible for μs-lived luminescence. This barrier limits the efficient population of the luminescent state at low temperatures, whereas at higher temperatures, the increased thermal energy is sufficient to overcome the barrier, thereby increasing the *I*_lu_/*I*_fl_ ratio.

**Table 2:** Time-Resolved Photophysical Properties of F1 and F2

sample	solvent	temp. (°C)	<τ>_ns_ ^a^	<τ>_μs_
F1	H_2_O	25	IRF^b^ (90 ps)^c^	22 μs
		–196	2,6 ns	327 μs
	D_2_O	25	IRF	72 μs
		–196	3,0 ns	560 μs
F2	H_2_O	25	IRF	67 μs
		–196	2,4 ns	258 μs
	D_2_O	25	IRF	155 μs
		–196	2,5 ns	434 μs

a <τ> is the intensity-weighted average decay time.

b IRF indicates that the decay time was too short to be determined with our TCSPC instrument.

c Value previously determined by fs-transient absorption measurements.^19^ Experimental details, together with the corresponding data can be found in the SI and in Figures S5–S8 and Tables S1 and S2.

On the other hand, in the case of F1, changes in the *I*_lu_/*I*_fl_ ratio cannot be discussed, since we have previously shown that most of the fluorescence appears to originate from the F2 emitter, which is present as an impurity.

### Mass spectrometry

Despite differences in the spectroscopic features, the mass spectra (Figure 3) of F1 and F2 are remarkably similar, exhibiting peaks at identical *m*/*z* values.

**Figure 3:**
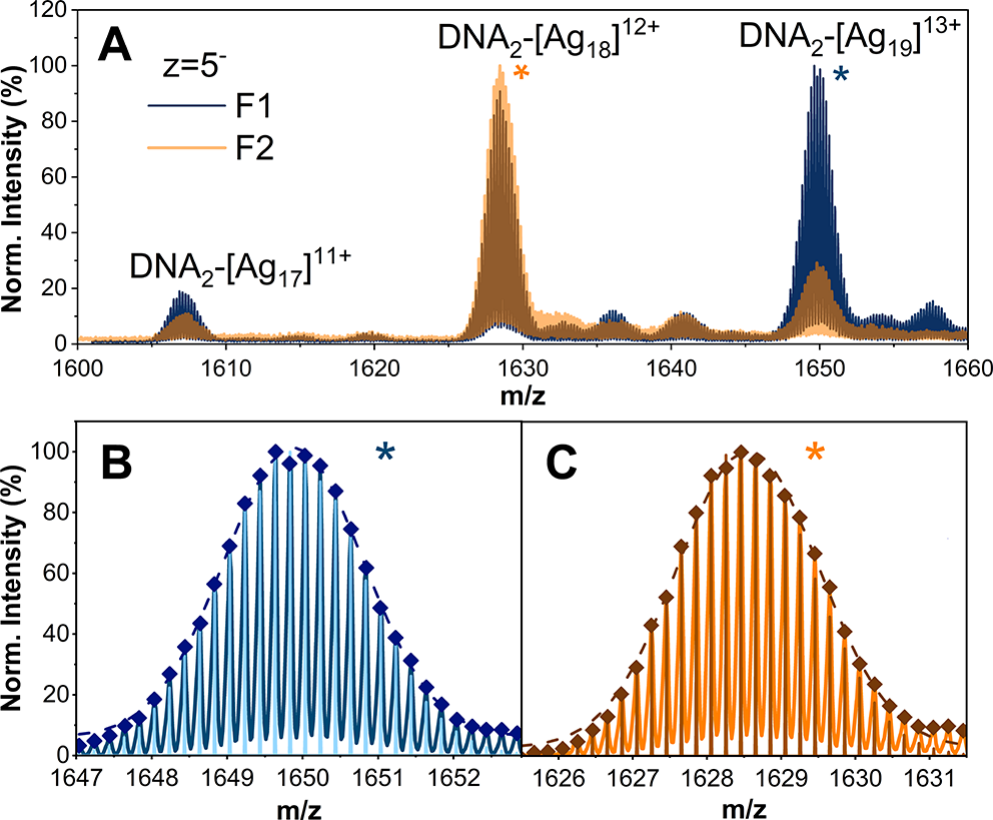
(A) Zoomed-in view of the mass spectra of F1 (blue) and F2 (orange) in the *z* = 5^–^ region. The full mass spectra are shown in Figure S12. (B) Peak of F1 assigned to DNA_2_[Ag_19_]^13+^. The experimental isotopic distribution (dark blue) is shown alongside the theoretical isotopic distribution (light blue) and Gaussian fit (dashed line). The calculated average mass is *m*/*z* 1649,881, which is consistent with the fully protonated molecular formula C_196_H_244_N_86_O_116_P_18_[Ag_19_]^13+^ minus 18 protons to reach a charge state of 5^–^. (C) Peak of F2 assigned to DNA_2_[Ag_18_]^12+^. The experimental isotopic distribution (orange) is reported together with the theoretical isotopic distribution (brown) and Gaussian fit (dashed line). The calculated average mass is *m*/*z* 1628,493, which corresponds to the fully protonated molecular formula C_196_H_244_N_86_O_116_P_18_[Ag_18_]^12+^ minus 17 protons to reach a charge state of 5^–^.

Specifically, for both F1 and F2, two peaks at *m*/*z* 2035,899 and 1628,511 for *z* = 4^–^ and *z* = 5^–^, respectively, corresponding to an experimental mass of 8159,74 g/mol, were observed. These peaks are consistent with a DNA-AgNC containing 18 silver atoms, of which 6 are neutral, resulting in an overall charge of 12+ (DNA_2_[Ag_18_]^12+^). This composition is in line with the previous analysis carried out by Rück et al.^19^ However, additional peaks at *m*/*z* 2062,584 and 1649,881 for *z* = 4^–^ and *z* = 5^–^, respectively, were found to match the mass of a DNA-AgNC composed of two DNA strands and 19 silver atoms with an overall charge of 13+. The primary distinction between F1 and F2 lies in the relative intensities of the peaks assigned to DNA_2_[Ag_18_]^12+^ and DNA_2_[Ag_19_]^13+^. For F1, the DNA_2_[Ag_19_]^13+^ peak dominates, whereas for F2, the DNA_2_[Ag_18_]^12+^ peak is more intense (see Figure 3A and Supporting Information for additional data analysis). In the mass spectrum of F2, the peak corresponding to DNA_2_[Ag_19_]^13+^ appears clearly in the *z* = 5^–^ charge state and is nearly absent at *z* = 4^–^, whereas the peak assigned to DNA_2_[Ag_18_]^12+^ remains dominant in both charge states (Figure S15). These mass spectrometry data demonstrate that the two fractions contain stoichiometrically identical species, but do not provide any information on the arrangement of silver atoms within the DNA-AgNCs. Given this limitation, it is paramount to complement mass spectrometry data with X-ray-based characterization methods to fully elucidate the structure and charge of DNA-AgNCs.

### Single Crystal X-ray Diffraction

Crystals of both species, F1 and F2, were grown using the hanging-drop vapor diffusion method at room temperature (see Figures S16 and S17). However, the dual-emissive cluster crystals were unstable, degrading rapidly under illumination during microscope inspection (Figure S16). Consequently, single-crystal X-ray diffraction data could only be obtained for F1, enabling its structural characterization. The atomic coordinates and experimental data have been deposited in the Protein Data Bank (PDB) with the accession code 23LP. The space group is *C*121, indicating a monoclinic crystal with lattice parameters *a* = 30,35 Å, *b* = 53,92 Å, *c* = 138,14 Å, and β = 89,97°. The asymmetric unit of this crystal is formed by three complete and two half DNA_2_[Ag_19_]^13+^ species (see Figure S18). Interestingly, unlike previously reported structures, there are no nucleobases that point away from the AgNC and facilitate crystal packing via π–π stacking interactions.^23,24^ As such, the DNA-AgNCs in the crystal rely solely on hydrogen bonds between nucleotides mediated by water molecules (Figures S19–S22). Three guanines, G_2_, G_9_, and G_10_, were identified to support these water-mediated interactions through their N1 atoms.

Moving from the crystal packing interactions to the structure of F1, Figure 4 illustrates the DNA conformation (panel A) and the arrangement of the silvers (Mol3 from Figure S18). The DNA_2_[Ag_19_]^13+^ cluster features two 10-base DNA strands and a central rod-like core composed of 17 silver atoms, flanked on both sides by two additional silver atoms. This peculiar construct will be then referred to as DNA_2_[Ag_17+2_]^13+^. For clarity, the two DNA strands in Figures 4 and 5 are colored green and blue, respectively. As shown in Figure 5A,B, these extra silvers are positioned at interatomic distances of 5,2 Å from the nearest atom in the AgNC rod. Each isolated silver binds to A_4_, C_5_ and G_6_ of the same strand. Specifically, G_6_ coordinates this silver via N7, A_4_ interacts through N3, while O2 is the binding site of C_5_. As mentioned previously, all nucleobases coordinate at least one silver. We even observe that thymine (T_1_) is involved in the stabilization of the AgNC. This is the first structural confirmation of a thymine, deprotonated at the N3 position, involved in an AgNC formation at pH 7. However, it has previously been shown that DNA strands composed solely of thymines can support the formation of AgNCs in a pH 10,5 buffer,^21^ while Perren et al. recently reported an Ag_4_ cluster located nearby a TT mismatch in tensegrity triangle tiles.^25^ Silver-induced deprotonation of nucleobases has also been investigated by David et al. using neutron scattering methods, further supporting the ability of silver species to modulate nucleobase protonation states.^26^

**Figure 4:**
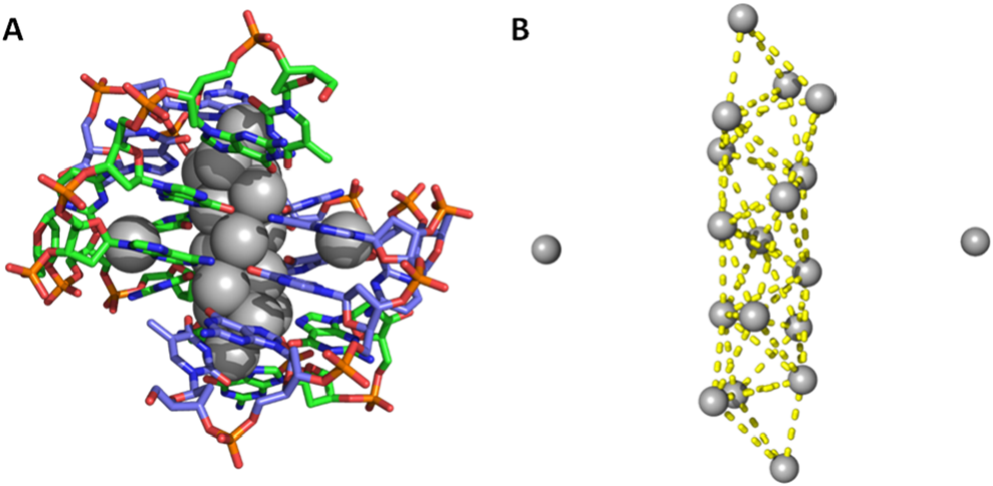
(A) Structure of DNA_2_[Ag_17+2_]^13+^ cluster. (B) Arrangement of the 19 silvers templated by the two decamers. The central AgNC consists of 17 silver atoms, organized in a rod-like geometry, and two additional silver atoms (most likely Ag^+^) on both sides of the elongated core. The yellow dotted lines indicate silver–silver interactions with a distance below 3,5 Å.

**Figure 5:**
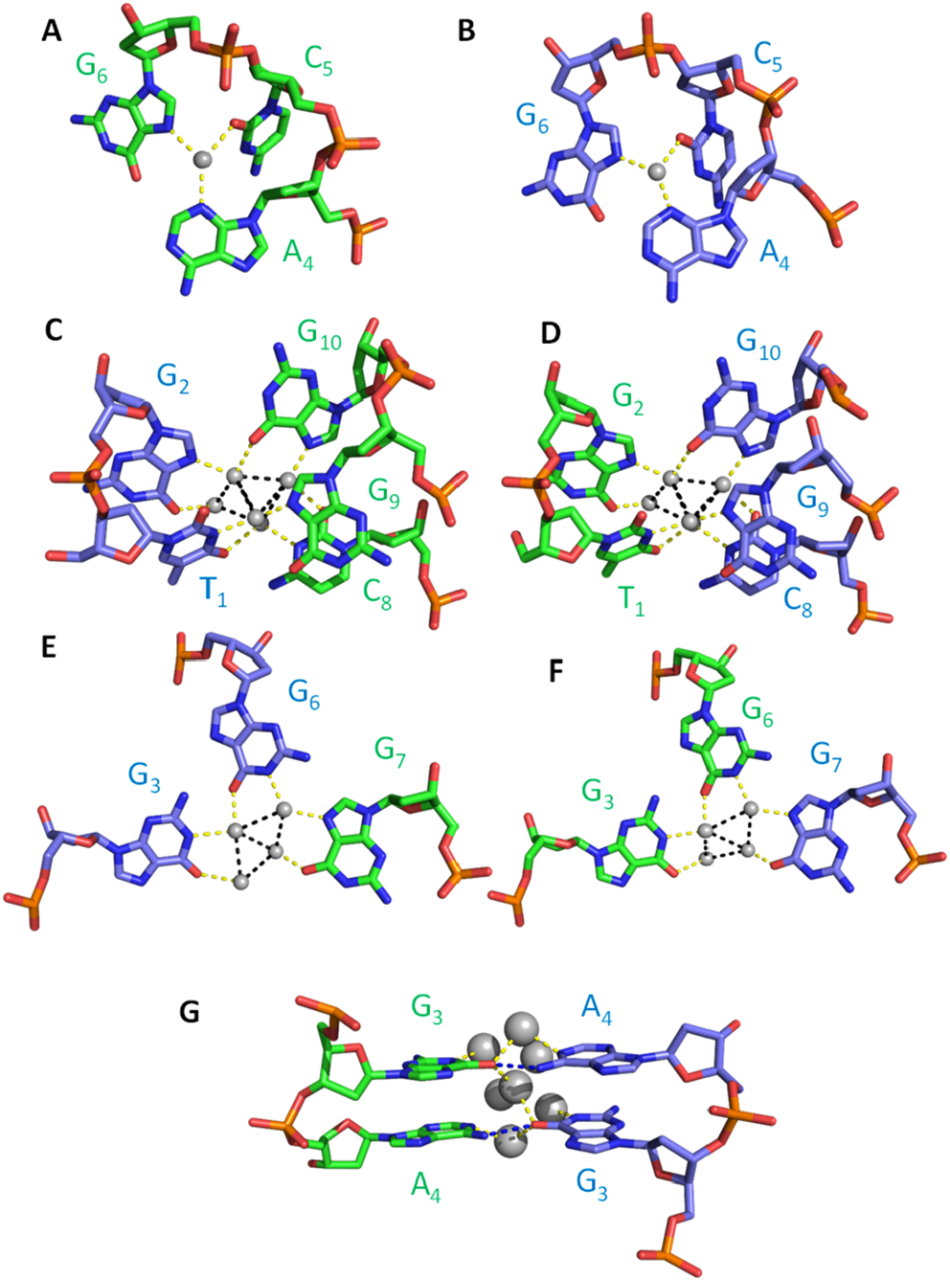
(A) and (B) interaction of the isolated silver atoms with A_4_, C_5_ and G_6_ of strand 1 (green) and strand 2 (blue). (C) and (D) top sections detailing the interactions of silvers with T1, and G2 of one strand and C_8_, G_9_ and G_10_ of the second strand. (E) and (F) middle sections detailing the interactions between silvers and G_3_, and G_6_ of one strand and G_7_ of the other strand. (G) side view of the middle section, showing how O5 of the two G_3_ nucleobases are located in between two silver positions.

Figure 5C shows that the T_1_ of the blue strand binds to two silvers through O2 and N3, which indicates that the thymine is deprotonated at position 3 (N···Ag distance: 2,2 Å). G_2_ (blue) binds to two silvers through N7 and O6, while the G_10_ base from the green strand interacts with two silvers via O6 and N7. Additionally, G_9_ (green) coordinates one silver through N7, while C_8_ (green) binds two silvers through O2 and N3. Figure 5D shows a similar interaction pattern, with inverted strand colors, on the other side of the DNA-AgNC. Next, Figure 5E illustrates that G_6_ (blue) binds two silvers through N1 and O6, whereas G_3_ (blue) coordinates one silver via N1 and two silvers through O6 (only one shown in Figure 5E, see Figure 5H for both interactions). Furthermore, G_7_ (green) binds to two silver atoms through O6 and N7. The same interactions can also be found back in Figure 5F with inverted strand colors. Interestingly, hydrogen bonds (depicted as blue dashed lines in Figure 5H) are formed between the O6 atom of G_3_ and one of the hydrogens attached to the N6 atom of A_4_ as the O···N distance is 2,7 Å. As a result, the O6 atom is positioned right in between two silver atoms and interacts with both (bond lengths are 2,4 and 2,6 Å). The N1 atom of G_6_ binds to the silver rod and acts as the bridge to the isolated silver atom that is coordinated by the N7 atom (Figure 5A).

### Single Crystal Photophysical Properties

Emission spectra of several crystals, grown using different conditions (description can be found in paragraph 7.1 of the SI), were recorded to confirm that the spectroscopic properties in the crystalline state are similar to those in solution.

Figure 6A illustrates examples of the purple DNA_2_[Ag_17+2_]^13+^ crystals, and Figure 6B shows an emission spectrum with a maximum located around 890 nm, resembling the emission spectrum of F1 in solution with a slight red-shift of ∼10 nm (see Figure 1C). Extra examples of emission spectra can be found in Figure S25A. Time-resolved measurements of the F1 crystals show luminescence decays with intensity-weighted lifetimes of 31–45 μs (Figure S25B). Compared to 22 μs for F1 in aqueous solution at 25 °C, the longer lifetimes in the crystalline state likely reflect reduced nonradiative decay due to the rigid crystal environment. The correspondence between the colors of both the DNA-AgNCs crystals and solution, along with the similar photophysical properties, confirms that the crystallization does not dramatically alter the solution structure of DNA_2_[Ag_17+2_]^13+^.

**Figure 6:**
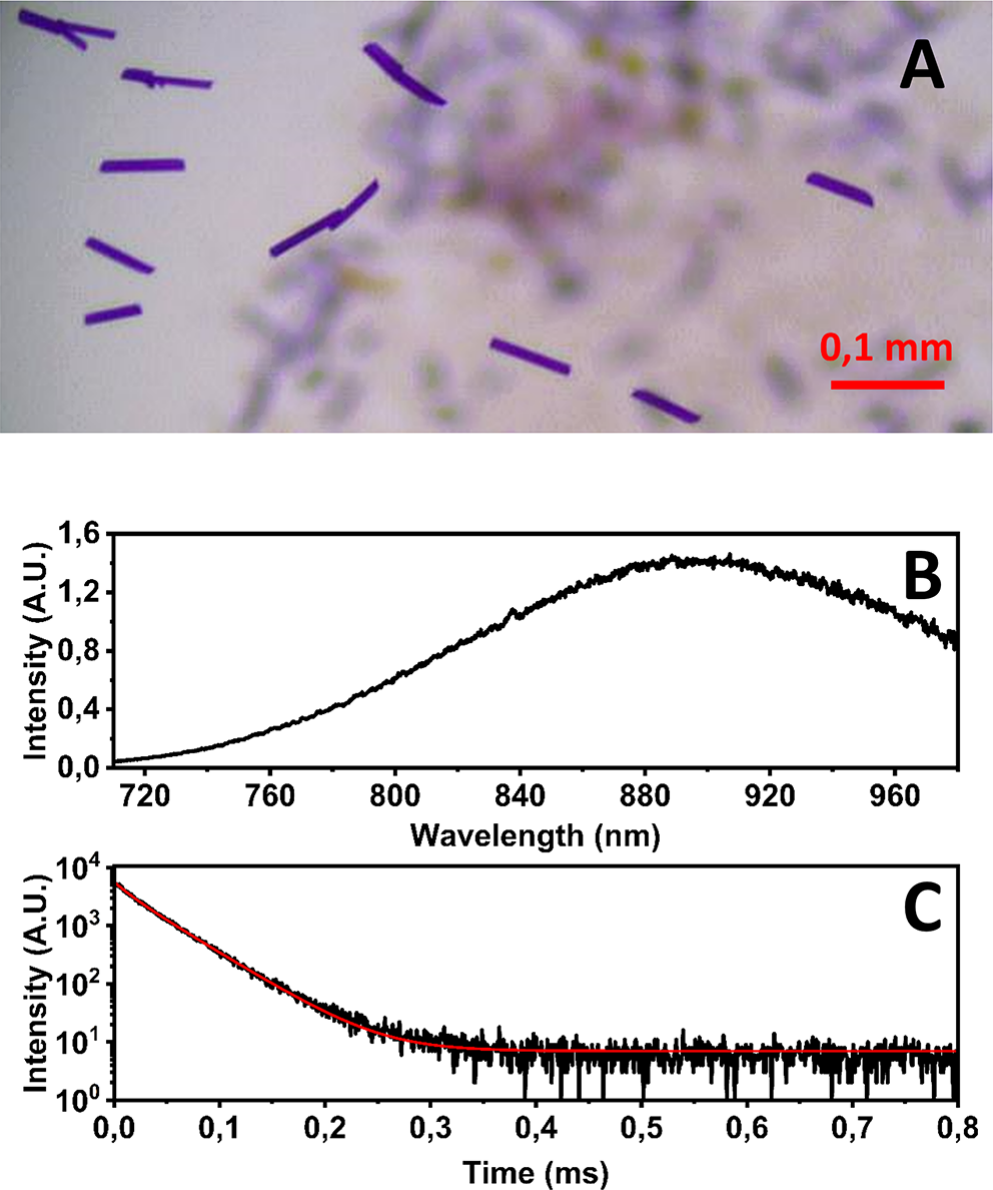
(A) Examples of purple DNA_2_[Ag_17+2_]^13+^ crystals. The crystals were grown in 10% polyethylene glycol (PEG) 3350, 10 mM spermine, 50 mM 3-(N-morpholino)­propanesulfonic acid (MOPS) with pH 7, and 200 mM NH_4_NO_3_. (B) Emission spectrum of a single DNA_2_[Ag_17+2_]^13+^ crystal. The spectrum was recorded on a home-built confocal microscope exciting at 520 nm. Additional spectra can be found in Figure S25A. (C) Luminescence decay of one crystal taken as example measured with a burst mode approach.^27^ The intensity-weighted average decay time ⟨τ⟩_μs_ was determined by tail-fitting the data with a biexponential function (red line). Fit values and additional decays can be found in Figure S25B.

## Conclusions

We have presented a new DNA-AgNC formed by a guanine-rich DNA sequence (5′-TGG ACG GCG G-3′), which stabilizes two closely related DNA-AgNCs with main absorption bands in the green range of the spectrum. The newly identified DNA_2_[Ag_17+2_]^13+^ species features a rod-like AgNC core composed of 17 silvers atoms, with one additional silver atom on each side of the longitudinal axis and exhibits predominantly microsecond-lived emission in solution. Mass spectrometry confirmed that 6 of those silver atoms are neutral while 13 are cationic. The crystal structure further revealed previously unobserved interactions, including coordinate bonds between thymines and the AgNC, water-mediated crystal packing interactions and O6 atoms of two guanines bridging two silver atoms. This structural insight provides a foundation for future studies aimed at understanding the origin of nanosecond- and microsecond-lived emission, as well as dual-emissive behavior in DNA-AgNCs. These results highlight DNA as a versatile scaffold with the appropriate balance between flexibility and rigidity allowing controlled access to a vast landscape of atomically precise silver nanoclusters. This structural and photophysical diversity will eventually enable rational design for future applications in sensing and bioimaging.

## Supplementary Material



## Data Availability

The data that support the findings of this study are available in the Supporting Information of this article and at 10.5281/zenodo.18746017.

## Notes

Synthesis and HPLC purification details, experimental details, steady-state and time-resolved emission data, mass-spectrometry data, and crystallization data (PDF)

## References

[1] Gonzàlez-Rosell A., Copp S. M. (2024). An Atom-Precise Understanding of DNA-Stabilized Silver
Nanoclusters. Acc. Chem. Res..

[2] Gonzàlez-Rosell A., Cerretani C., Mastracco P., Vosch T., Copp S. M. (2021). Structure
and luminescence of DNA-templated silver clusters. Nanoscale Adv..

[3] Choi S., Dickson R. M., Yu J. (2012). Developing
luminescent silver nanodots
for biological applications. Chem. Soc. Rev..

[4] O’Neill P. R., Young K., Schiffels D., Fygenson D. K. (2012). Few-Atom Fluorescent
Silver Clusters Assemble at Programmed Sites on DNA Nanotubes. Nano Lett..

[5] Petty J. T., Story S. P., Juarez S., Votto S. S., Herbst A. G., Degtyareva N. N., Sengupta B. (2012). Optical Sensing by Transforming Chromophoric
Silver Clusters in DNA Nanoreactors. Anal. Chem..

[6] Yeh H.-C., Sharma J., Shih I.-M., Vu D. M., Martinez J. S., Werner J. H. (2012). A Fluorescence Light-Up
Ag Nanocluster Probe That Discriminates
Single-Nucleotide Variants by Emission Color. J. Am. Chem. Soc..

[7] Guha R., Gonzàlez-Rosell A., Rafik M., Arevalos N., Katz B. B., Copp S. M. (2023). Electron
count and ligand composition
influence the optical and chiroptical signatures of far-red and NIR-emissive
DNA-stabilized silver nanoclusters. Chem. Sci..

[8] Petty J. T., Zheng J., Hud N. V., Dickson R. M. (2004). DNA-Templated Ag
Nanocluster Formation. J. Am. Chem. Soc..

[9] Copp S. M., Schultz D., Swasey S., Pavlovich J., Debord M., Chiu A., Olsson K., Gwinn E. (2014). Magic Numbers
in DNA-Stabilized Fluorescent Silver Clusters Lead to Magic Colors. J. Phys. Chem. Lett..

[10] Malola S., Häkkinen H. (2025). Tuning the
electronic structure of a rod-like DNA-stabilized
silver nanocluster Ag28Cl2 for photophysics in the NIR-II window. Chem. Commun..

[11] Malola S., Matus M. F., Häkkinen H. (2023). Theoretical
Analysis of the Electronic
Structure and Optical Properties of DNA-Stabilized Silver Cluster
Ag16Cl2 in Aqueous Solvent. J. Phys. Chem. C.

[12] Ramazanov R. R., Nasibullin R. T., Sundholm D., Kurtén T., Valiev R. R. (2024). Nonradiative Deactivation of the Fluorescent Ag16-DNA
and Ag10-DNA Emitters: The Role of Water. J.
Phys. Chem. Lett..

[13] Ramazanov R. R., Valiev R. R. (2025). How Water Regulates
Nonradiative Deactivation of DNA-Templated
Green-Yellow Rod-Shaped Silver Clusters. J.
Phys. Chem. B.

[14] Zhai F., Guan Y., Li Y., Chen S., He R. (2022). Predicting
the Fluorescence Properties of Hairpin-DNA-Templated Silver Nanoclusters
via Deep Learning. ACS Appl. Nano Mater..

[15] Müller J. (2019). Nucleic acid
duplexes with metal-mediated base pairs and their structures. Coord. Chem. Rev..

[16] López-Chamorro C., Pérez-Romero A., Domínguez-Martín A., Javornik U., Palacios O., Plavec J., Galindo M. A. (2025). Silver
Binding Dichotomy for 7-Deazaadenine/Thymine: Preference for Watson–Crick
Pairing over Homobase Interactions in DNA. Inorg.
Chem..

[17] Ritchie C. M., Johnsen K. R., Kiser J. R., Antoku Y., Dickson R. M., Petty J. T. (2007). Ag Nanocluster Formation Using a Cytosine Oligonucleotide
Template. J. Phys. Chem. C.

[18] Schultz D., Gwinn E. G. (2012). Silver atom and
strand numbers in fluorescent and dark
Ag:DNAs. Chem. Commun..

[19] Rück V., Liisberg M. B., M?llerup C. B., He Y., Chen J., Cerretani C., Vosch T. (2023). A DNA-Stabilized Ag1812+
Cluster
with Excitation-Intensity-Dependent Dual Emission. Angew. Chem., Int. Ed..

[20] Mastracco P., Gonzàlez-Rosell A., Evans J., Bogdanov P., Copp S. M. (2022). Chemistry-Informed Machine Learning Enables Discovery
of DNA-Stabilized Silver Nanoclusters with Near-Infrared Fluorescence. ACS Nano.

[21] Sengupta B., Ritchie C. M., Buckman J. G., Johnsen K. R., Goodwin P. M., Petty J. T. (2008). Base-Directed Formation
of Fluorescent Silver Clusters. J. Phys. Chem.
C.

[22] Maillard J., Klehs K., Rumble C., Vauthey E., Heilemann M., Fürstenberg A. (2021). Universal quenching of common fluorescent probes by
water and alcohols. Chem. Sci..

[23] Cerretani C., Kanazawa H., Vosch T., Kondo J. (2019). Crystal structure of
a NIR-Emitting DNA-Stabilized Ag16 Nanocluster. Angew. Chem., Int. Ed..

[24] Romolini G., Kanazawa H., M?llerup C. B., Liisberg M. B., Lind S. W., Huang Z., Cerretani C., Kondo J., Vosch T. (2025). Shining Bright
at 960 nm: A 28-Silver-Atom Nanorod Stabilized by DNA. Small Struct..

[25] Perren, L. ; Livernois, W. ; Woloszyn, K. ; Janowski, J. ; Faiaz, L. ; Singh, V. R. ; Jaffe, M. ; Mao, C. ; Canary, J. W. ; Ohayon, Y. P. ; Sha, R. ; Anantram, M. P. ; Vecchioni, S. Topology-Enforced Synthesis of Atomically-Precise Silver Nanoclusters in 3D DNA Lattices. 2025 10.26434/chemrxiv-2025-3f6br-v2.

[26] David F., Setzler C., Sorescu A., Lieberman R. L., Meilleur F., Petty J. T. (2022). Mapping H+ in the Nanoscale (A2C4)­2-Ag8
Fluorophore. J. Phys. Chem. Lett..

[27] Liisberg M. B., Krause S., Cerretani C., Vosch T. (2022). Probing emission of
a DNA-stabilized silver nanocluster from the sub-nanosecond to millisecond
timescale in a single measurement. Chem. Sci..

