# Supplementary material for: Two of a Kind: Composition and Photophysics of Two Silver Nanoclusters Stabilized by the Same DNA Sequence
Source: J Am Chem Soc. 2026 Jun 18;148(25):25989–95. doi: 10.1021/jacs.6c04812 (PMC13340420; doi:10.1021/jacs.6c04812)
Supplement: Supplementary file 1 [file ja6c04812_si_001.pdf]

Supplementary Information.

Two of a Kind: Composition and Photophysics of Two Silver Nanoclusters Stabilized by the Same DNA Sequence

Letizia Liccardo,[a,1] Hiroki Kanazawa,[b,1] Giacomo Romolini,[a] Cecilia Cerretani,[a] Simon Wentzel Lind,[a] Beatrice Polido,[c] Christian Brinch Mollerup,[d] Vanessa Rück,[a] Zhiyu Huang,[a] Leila Lo Leggio,[a] Jiro Kondo,[b,*] and Tom Vosch[a,*]

[a] Letizia Liccardo, Simon Wentzel Lind, Vanessa Rück, Giacomo Romolini, Cecilia Cerretani, Tom Vosch
Department of Chemistry
University of Copenhagen
Universitetsparken 5, 2100 Copenhagen, Denmark.
E-mail: tom@chem.ku.dk

[b] Hiroki Kanazawa, Jiro Kondo
Department of Materials and Life Sciences,
Sophia University, 7-1 Kioi-cho, Chiyoda-ku, 102-8554 Tokyo, Japan.
E-mail: j.kondo@sophia.ac.jp

[c] Beatrice Polido
Department of Molecular Sciences and Nanosystems,
Ca' Foscari University of Venice, Via Torino 155, 30170 Mestre, Venezia, Italy.

[d] Christian Brinch Mollerup
Department of Forensic Medicine
University of Copenhagen
Frederik V's Vej 11, 2100 Copenhagen, Denmark.

Materials and Methods

1. Materials

The DNA oligonucleotide and nuclease-free water were purchased from Integrated DNA Technologies. $AgNO_3$ (≥ 99.998%), $NaBH_4$ (≥ 99.99%), and ammonium acetate (≥ 98%) were purchased from Sigma Aldrich.

2. Synthesis

Both DNA-AgNCs were synthesized in the same reaction mixture, following to the protocol reported by Rück *et al.*[1] Briefly, hydrated DNA oligomers with the sequence 5'-TGG ACG GCG G-3' were mixed with $AgNO_3$ in a 10 mM ammonium acetate (NH_4OAc) aqueous solution at pH 7. Freshly prepared $NaBH_4$ was added to the mixture after 15 min. The ratio between the components [DNA]:[$AgNO_3$]:[$NaBH_4$] was 30 µM: 150 µM: 75 µM. After synthesis, the sample was stored in the fridge for 6 days prior to high-performance liquid chromatography (HPLC) purification. Finally, the two purified fractions were solvent exchanged to 10 mM NH_4OAc H_2O solution by spin-filtration with a 3 kDa cut-off membrane filter (Amicon Ultracel-3).

3. HPLC purification

HPLC purification was performed using a preparative HPLC system from Agilent Technologies with an Agilent Technologies 1100 Series UV-Vis detector, an Agilent Technologies 1260 Infinity fluorescence detector and a LUNA C18 column (5 µm, 100 Å, 250 × 10 mm, Phenomenex), equipped with a fraction collector. The mobile phase was a gradient mixture of 35 mM triethylammonium acetate (TEAA) buffer in water (A) and methanol (B). The flow rate was set to 4.7 mL/min.

The elution gradient ranged from 10% to 95% B in 30 min. In the first 2 min it was kept constant at 10% B, then it was linearly increased to 30% B in the next 20 min, and finally in the 22-25 min interval, the gradient was rapidly risen to 95% B.

The collection was based on the AgNCs absorbance at 540 nm, while monitoring also the DNA absorbance at 260 nm and the absorption of silver nanoparticles (λ = 450 nm, not shown). At the same time, both AgNCs emission bands were monitored at 630 and 830 nm, exciting at 540 nm.

The run was followed by 5 min of washing with 95% B to remove any remaining sample from the column.

As shown in the chromatograms in Figure 1, the two purified fractions, corresponding to F1 and F2, eluted around 8.7 and 14.2 min, *i.e.*, approximately 17% and 22% B.

4. Spectroscopic measurements in solution

Steady-state and time-resolved measurements were carried out in 10 mM NH_4OAc H_2O and D_2O solutions, at both -196 °C, and 25 °C. A description of the measurements at -196 °C is given in paragraph 4.5. For cuvette measurements, samples in a 10 mM NH_4OAc D_2O solution were prepared by adding 5-10 µL of concentrated DNA-AgNC stock solution (synthesized in an H_2O environment) to 2-3 mL of the solvent. For microscope measurements, described in Section 4.3, a droplet of 10-20 µL of sample was deposited onto a coverslip.

4.1 Absorption measurements

Absorption spectra were measured with a Lambda 800 UV/Vis spectrophotometer from Perkin Elmer using a deuterium lamp for ultraviolet radiation and a tungsten-halogen lamp for visible radiation. All measurements were performed in a single-beam configuration with a "zero/baseline" correction, *i.e.*, measuring the 100%/0% transmittance with air as reference. The corresponding solvent spectra were measured separately and then subtracted from the samples' spectra. The absorbance of the samples was kept below 0.1 to avoid inner filter effects during emission measurements.

4.2 Steady-state emission measurements

Steady-state fluorescence measurements were performed using either a QuantaMaster 400 Fluorometer (Horiba) equipped with a Xenon arc lamp or a FluoTime300 instrument (PicoQuant). The fluorescence spectra acquired with the Horiba fluorometer were measured by exciting at different wavelengths (350, 460, 530 and 630 nm) with an integration time of 0.2 s. Whereas the fluorescence spectra recorded with FluoTime300 were obtained by exciting the samples with a vertically-polarized 531 nm (LDH-D-TA-530B) picosecond-pulsed laser (PicoQuant). All emission spectra have been corrected for the wavelength dependency of the detector.

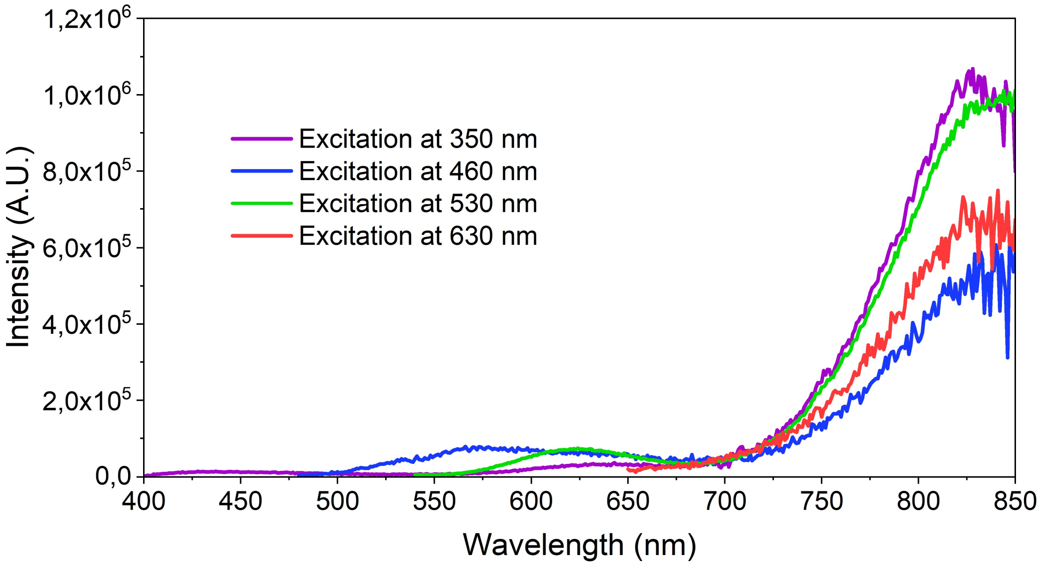

Figure S1. Emission spectra of F1 in 10 mM NH_4OAc aqueous solution at room temperature, monitoring the excitation at 350, 460, 530 and 630 nm. The measurements were performed using a QuantaMaster 400 Fluorometer (Horiba) equipped with Xenon arc lamp.

4.3 Steady-state emission measurements on the home-built microscope setup

The detector of the FluoTime300 system (PicoQuant), used for most measurements, becomes increasingly insensitive beyond 800 nm. To circumvent the low efficiency in the NIR range and to obtain emission spectra with more accurate spectral profiles, we used our home-built confocal microscope for measuring representative emission spectra of both F1 and F2 in 10 mM NH_4OAc aqueous solutions. These spectra are presented in Figure 1.

In detail, these measurements were performed by putting a droplet of solution on top of a glass coverslip staged on a microscope (Olympus IX71).[2] A continuum white-light laser (SuperK EXTREME EXB-6, NKT Photonics) was used as an excitation source delivering a wavelength of 514 nm by sending the continuum output through an acousto-optic tunable filter (SuperK SELECT, NKT Photonics). The output of the laser was expanded and collimated by a lens system and cleaned up by several filters (LL01-514-25, FF01-520/5-25, and FF01-650/SP-25, Semrock) before it was reflected by a 30:70 beam splitter (XF122, Omega Optical) and sent through an oil objective (100x, NA= 1.4, Olympus). Excitation power on top of the sample was 160 nW. The objective that collected the fluorescence was directed through a 100 µm pinhole and a 514 nm long-pass filter (LP02-514RE-25, Semrock). The fluorescence was then sent through a spectrograph (SP 2356 spectrometer, 300 grooves/mm, Acton Research) onto a nitrogen cooled CCD camera (SPEC-10:100B/LN-eXcelon, Princeton Instruments) for the recording of the spectra. Finally, the emission spectra were wavelength and intensity corrected as reported previously.[2]

4.4 Quantum Yield (ϕ) measurements and calculations

The luminescence quantum yield of F1 was determined in a 10 mM NH_4OAc aqueous solution at 25 °C, using Cresyl Violet in ethanol as reference dye.[3] Absorption and emission spectra of the DNA-AgNC and the reference compound were measured at five different concentrations (Figures S2 and S3).

In order to determine the luminescence quantum yield of F1, the emission spectra were corrected for the limited sensitivity of the FluoTime300 hybrid detector above 800 nm and the minor contribution of the F2 impurity in the F1 emission (Figure S2D). First, the emission spectra of F1 were corrected by using the emission spectrum measured on the home-built microscope (see section 4.2 and Figure 1C) rescaled to the 700 - 800 nm slope that was reliably measured on the FluoTime 300. Then, to account for the F2 contribution in the F1 emission, the emission spectra were further corrected by subtracting a scaled F2 spectrum from the F1 emission (Figure S2B). The amount of F2 was estimated by dividing the integrated area of ‘F2 scaled’ by ‘F1’, corrected for their respective fluorescence quantum yields (0.02 and 0.016), under the assumption that the excitation probability for F1 and F2 are similar. This yielded an estimated contribution of F2 to the total emission of approximately 3.5 %.

The integrated area of the corrected luminescence peaks was then plotted against the fraction of absorbed light, $1\text{-}10^{-Abs}$, at the excitation wavelength (531 nm) for every concentration. The same procedure was applied to the reference dye. Finally, the data were fitted linearly with the y-intercept fixed at zero, and the slopes were used to calculate the luminescence quantum yield of F1 according to the following equation:[4]

$$\phi_{NC} = \frac{Slope_{NC}}{Slope_{ref}} \cdot \frac{n_{NC}^2}{n_{ref}^2} \cdot \phi_{ref} \qquad \textbf{(1)}$$

where ϕ_{ref} represents the quantum yield of the reference (0.56)[3], and *n* is the refractive index of the solvent in which the compounds were dissolved during the measurements. The subscripts *NC* and *ref* indicate the DNA-AgNC (*i.e.*, F1 sample) and Cresyl Violet, respectively.

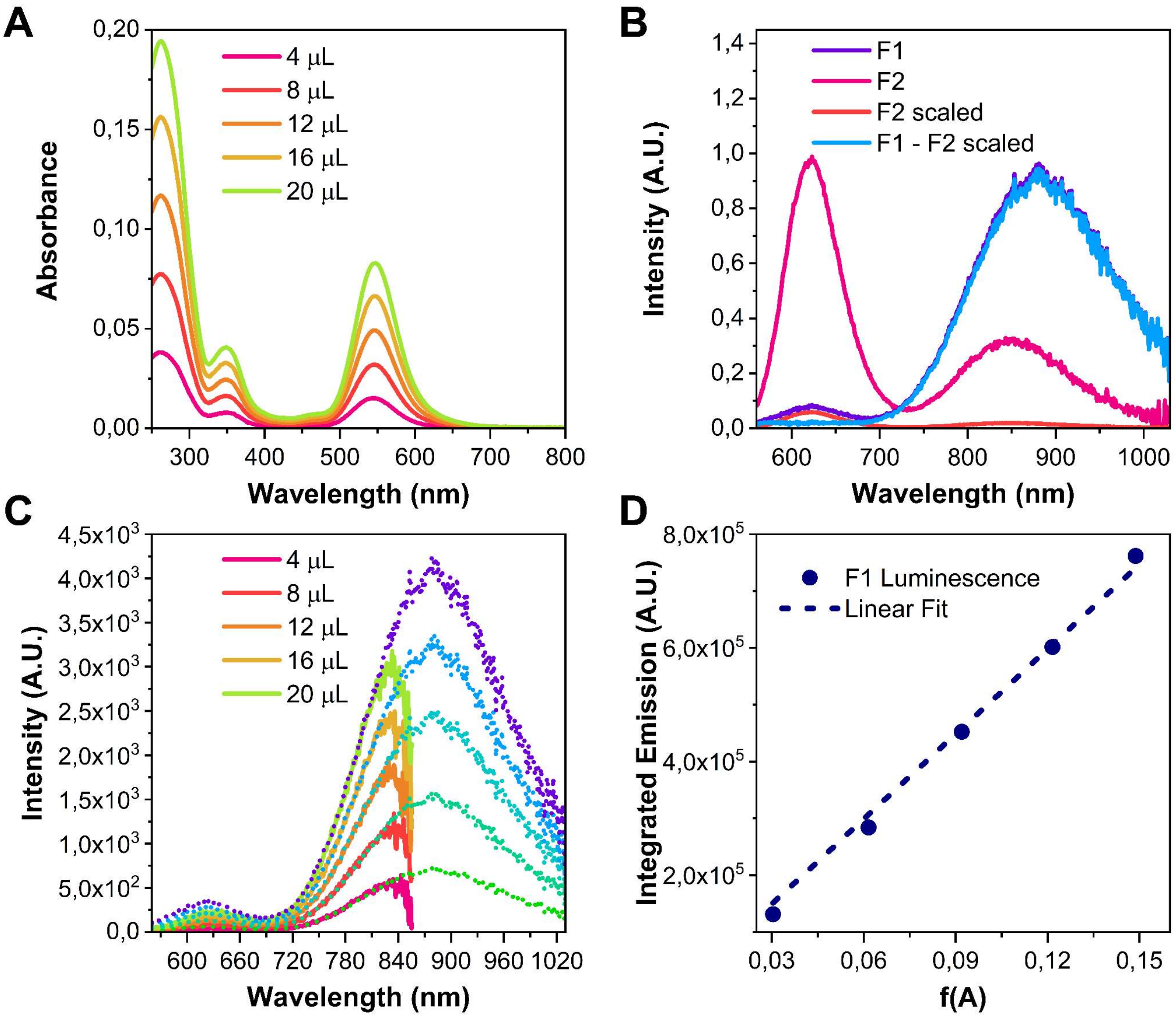

Figure S2. Quantum Yield data for F1. **(A)** Absorption spectra; **(B) e**xample of subtraction of the F2 contribution from the F1 emission (F1-F2 scaled, light blue), where F2 is rescaled (red) to match the fluorescence maximum at 620 nm in the F1 spectrum (purple); **(C)** corrected emission spectra (dashed lines); **(D)** linear fit of the integrated areas as a function of the fraction of absorbed light for F1 (note that this is F1 – F2 scaled from Figure S2B, and only in the spectral range from 700 nm to 1030 nm) in 10 mM NH_4OAc aqueous solution at 25 °C. The emission spectra were recorded by exciting at 531 nm.

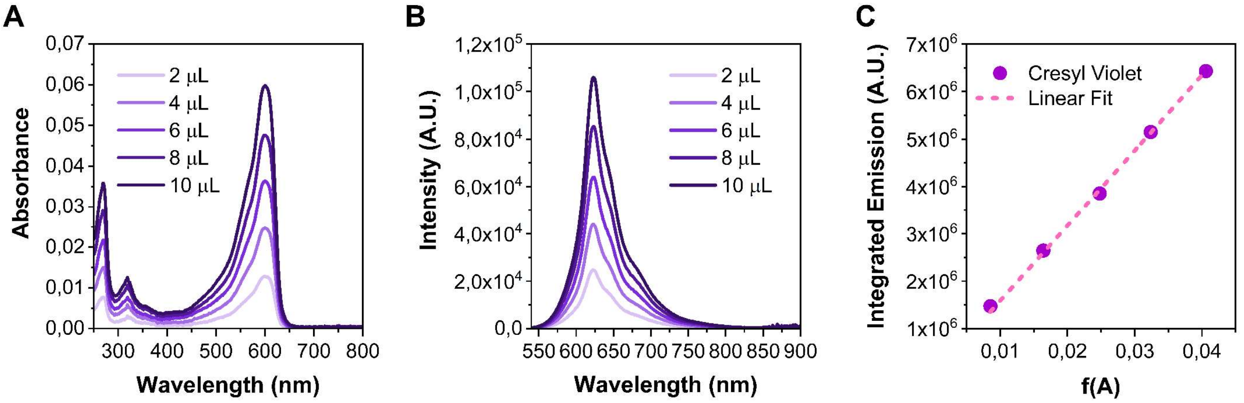

Figure S3. Quantum yield data for the reference dye. **(A)** Absorption spectra, **(B)** emission spectra and **(C)** linear fit of the integrated areas as a function of the fraction of absorbed light for the reference dye, Cresyl Violet, in ethanol, at 25 °C. The emission spectra were recorded by exciting at 531 nm.

4.5 Excitation spectra measurements

Excitation spectra were recorded with a QuantaMaster 400 instrument from Horiba with a Xenon arc lamp, monitoring the emission at 620 and 800 nm. The spectra were then corrected for the wavelength dependency of the detector and for the wavelength-dependent intensity difference of the excitation lamp.

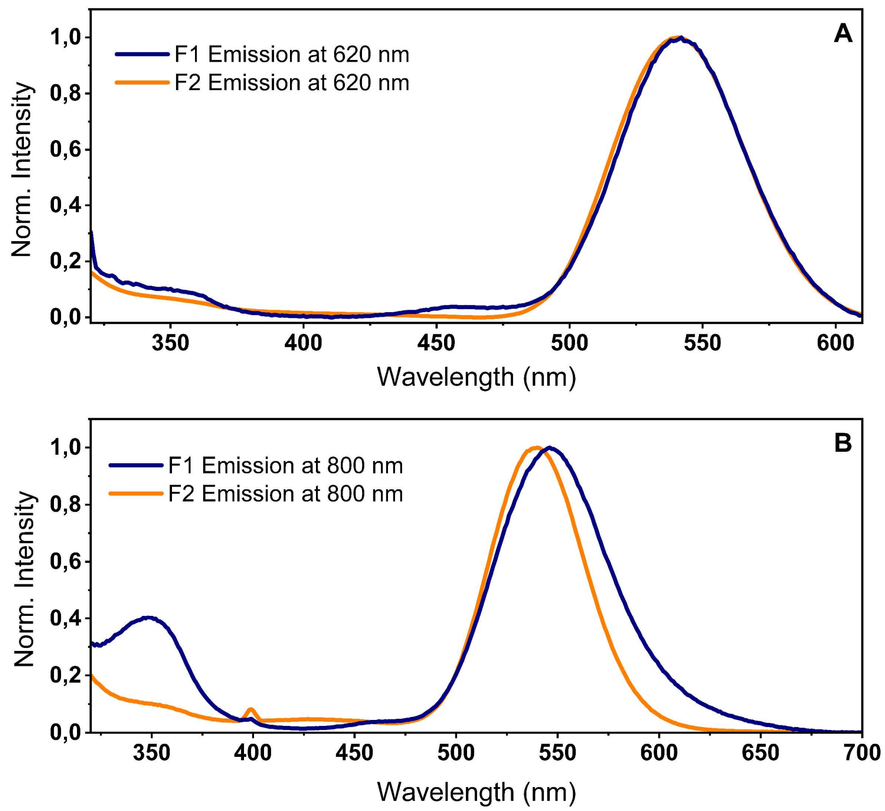

Figure S4. Comparison between the excitation profiles of F1 (blue) and F2 (orange) in 10 mM NH_4OAc aqueous solution at 25 °C. Normalized excitation spectra monitoring the emission at **(A)** 620 nm and **(B)** 800 nm.

4.6 Time-correlated single photon counting (TCSPC) measurements

Time-resolved fluorescence measurements were performed on a FluoTime 300 instrument (PicoQuant) using a vertically polarized 531 nm picosecond-pulsed laser (LDH-D-TA-530B, PicoQuant) for excitation.

For F1 and F2, fluorescence decays were acquired at 600 or 620 nm, integrating 5 s in order to reach at least 10^4 counts in the maximum. The decay curves measured in 10 mM NH_4OAc H_2O and D_2O solutions at 25 °C were found to be instrument response function (IRF)-limited (Table 2), thus it was not possible to determine

the fluorescence lifetimes in these conditions. For all other conditions at -196 °C, the decays were fitted with FluoFit v.4.6 software from PicoQuant, using a bi-exponential reconvolution model including the IRF. The average lifetimes $<\tau>_{ns}$, reported in Table 2, were calculated as the intensity-weighted average decay times at the monitored wavelength.[4]

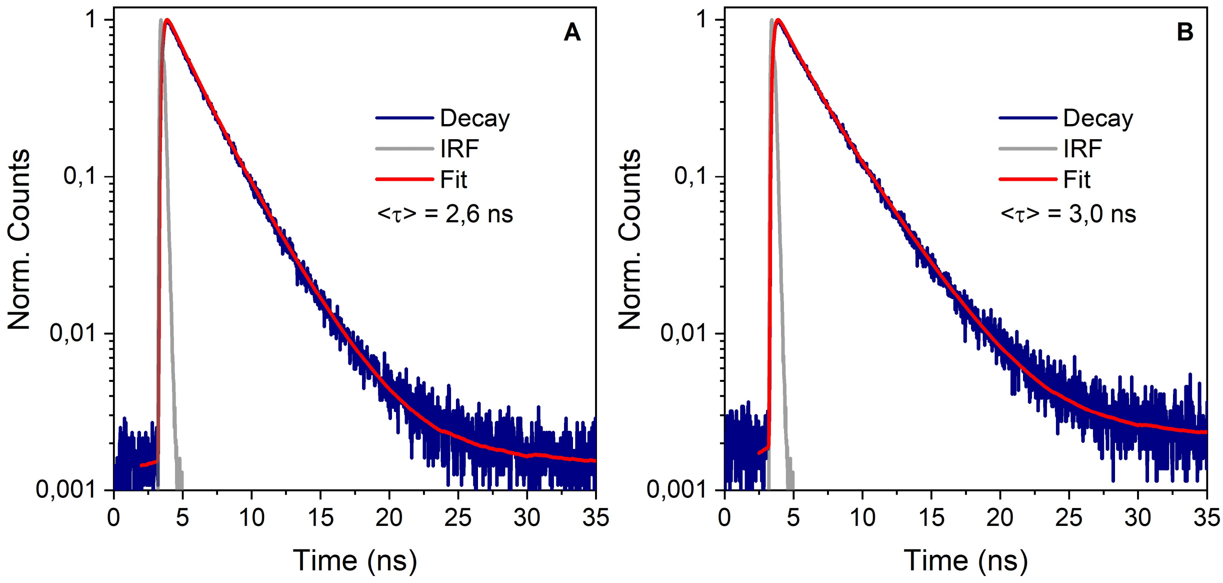

Figure S5. Normalized nanosecond decay curves (blue) of F1 in **(A)** 10 mM NH_4OAc H_2O solution and **(B)** 10 mM NH_4OAc D_2O solution, both at -196 °C, including bi-exponential fits (red), and the IRFs (gray). The decays were monitored at 600 nm, exciting at 531 nm.

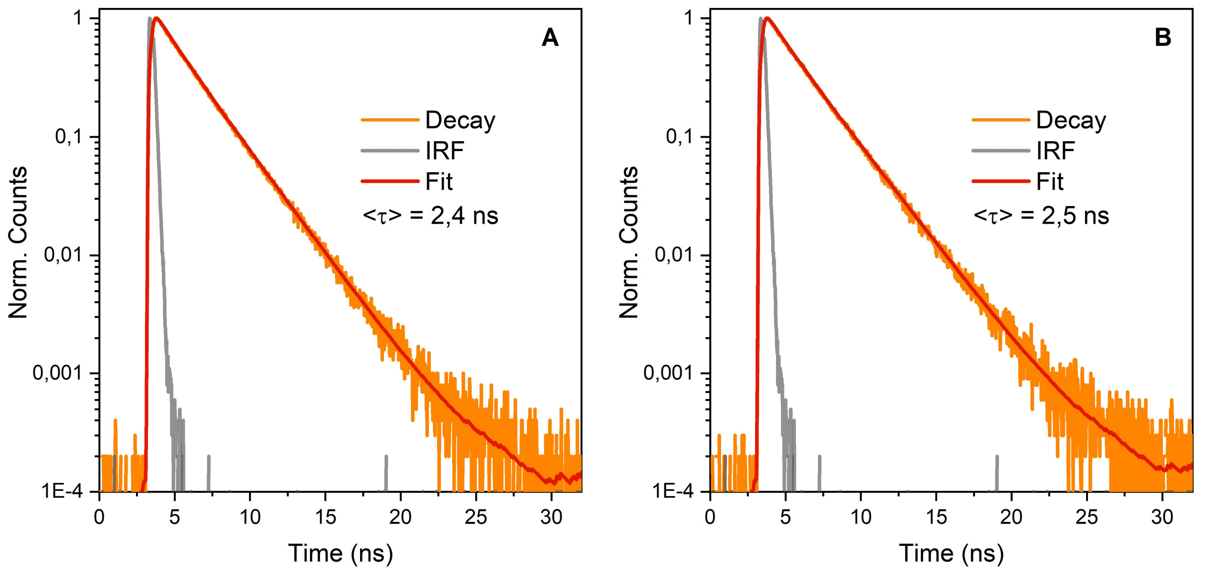

Figure S6. Normalized nanosecond decay curves (orange) of F2 in **(A)** 10 mM NH_4OAc H_2O solution and **(B)** 10 mM NH_4OAc D_2O solution, both at -196°C, including bi-exponential fits (red), and the IRFs (gray). The decays were monitored at 600 nm, exciting at 531 nm.

4.7 Microsecond decay time measurements

Microsecond decay times were measured with a burst mode approach on a FluoTime 300 instrument (PicoQuant) using a vertically polarized 531 nm picosecond-pulsed laser (LDH-D-TA-530B, PicoQuant) for excitation. Emission intensities of F1 and F2 in 10 mM NH_4OAc H_2O or D_2O solutions were recorded at 800 nm at both -196 °C and 25 °C. The integration time was set to 10 min. Every solvent and temperature condition required different settings that are summarized in Tables S1 and S2.

Table S1. *Settings for luminescence measurements of F1 in 10 mM NH_4OAc aqueous and deuterated solutions at 25 °C and -196 °C.*

Condition	Repetition rate	Effective sync rate	Burst length	# pulses	Period length	Duty cycle
H_2O 25 °C	40 MHz	1 kHz	400 µs	16.000	1,00 ms	40 %
D_2O 25 °C	40 MHz	100 Hz	4 ms	160.000	10 ms	40 %
H_2O -196 °C	40 MHz	500 Hz	750 µs	30.000	2,00 ms	37,5 %
D_2O -196 °C	40 MHz	100 Hz	3,75 ms	150.000	10,0 ms	37,5 %

Table S2. *Settings for luminescence measurements of F2 in 10 mM NH_4OAc aqueous and deuterated solutions at 25 °C and -196 °C.*

Condition	Repetition rate	Effective sync rate	Burst length	# pulses	Period length	Duty cycle
H_2O 25 °C	40 MHz	250 Hz	1,25 ms	50.000	4,00 ms	31,25%
D_2O 25 °C	40 MHz	250 Hz	1,25 ms	50.000	4,00 ms	31,25%
H_2O -196 °C	40 MHz	100 Hz	3,0 ms	120.000	10,0 ms	30%
D_2O -196 °C	40 MHz	100 Hz	3,0 ms	120.000	10,0 ms	30%

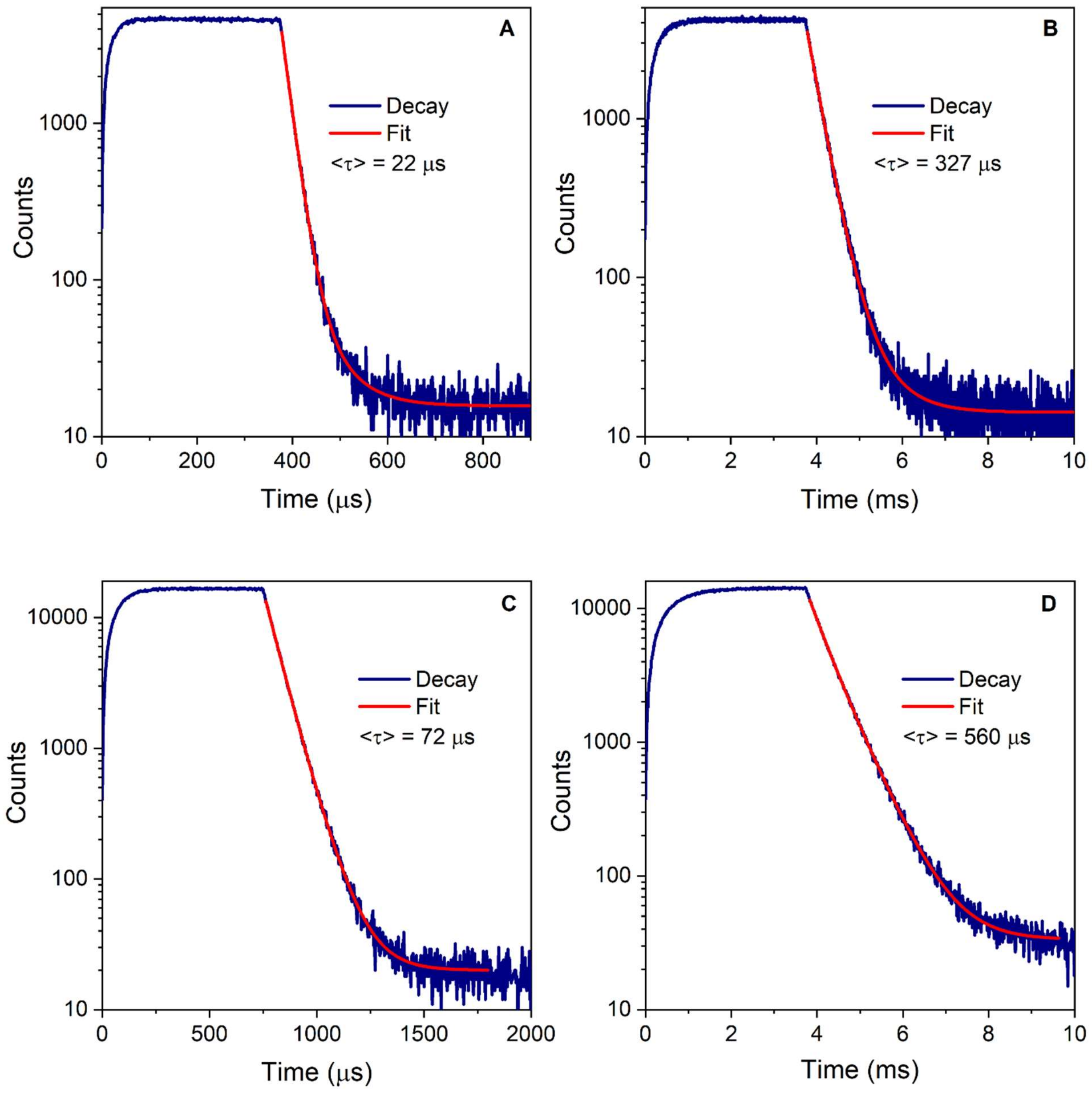

Figure S7. Luminescence decays of F1 measured with a burst mode approach. **(A-B)** Sample in 10 mM NH_4OAc aqueous solution at 25 °C and -196 °C, respectively. **(C-D)** Sample in 10 mM NH_4OAc D_2O solution at 25 °C and -196 °C, respectively. The emission was monitored at 800 nm, exciting at 531 nm.

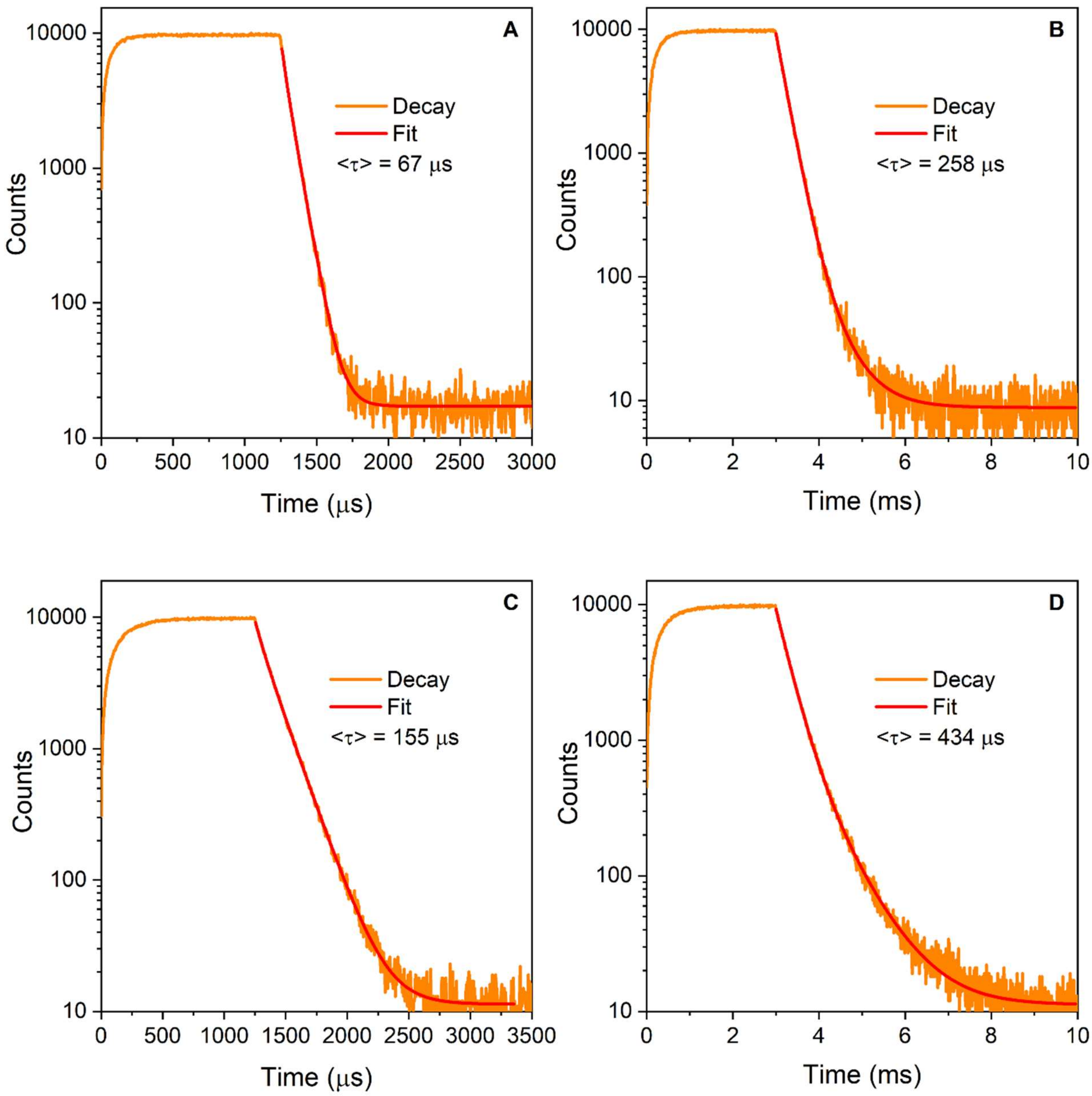

Figure S8. Luminescence decays of F2 measured with a burst mode approach. **(A-B)** Sample in 10 mM NH_4OAc aqueous solution at 25 °C and -196 °C, respectively. **(C-D)** Sample in 10 mM NH_4OAc D_2O solution at 25 °C and -196 °C, respectively. The emission was monitored at 800 nm, exciting at 531 nm.

4.8 Steady-state and time-resolved emission measurements at -196 °C

Low-temperature steady-state and time-resolved emission measurements were carried out in both 10 mM NH_4OAc H_2O and D_2O solutions. The measurements were performed by immersing an NMR tube with the sample in a transparent Dewar filled with liquid nitrogen (-196 °C). The Dewar was then placed in the cuvette compartment of a FluoTime300 instrument from PicoQuant. In order to limit the increased scattering, two filters were used: a 527 nm band-pass filter (Semrock, FF01-527/20-25) in the excitation path and a 532 nm long-pass filter (Semrock, BLP01-532R-25) in the emission path.

The acquired steady-state emission spectra were corrected for the wavelength dependency of the detector.

Fluorescence decay curves were measured at 600 nm, given the blue-shift of the emission spectrum at -196 °C. The analysis of time-resolved data was performed with FluoFit v.4.6 software from PicoQuant. The decays were fitted with a bi-exponential reconvolution model including the IRF. The intensity-averaged decay

times <τ>ns (reported in Table 1) were calculated as the intensity-weighted average decay times at the selected emission wavelength.[4]

Burst mode measurements were carried out by monitoring the emission at 800 nm using different settings for the two solvents (Tables S1 and S2).

The intensity decays upon switching off the laser were tail-fitted with a bi-exponential function, and intensity-weighted average luminescence decay times, $\langle\tau\rangle_{\mu s}$, are reported in Table 2, whereas the intensity traces can be found in Figures S7 and S8.

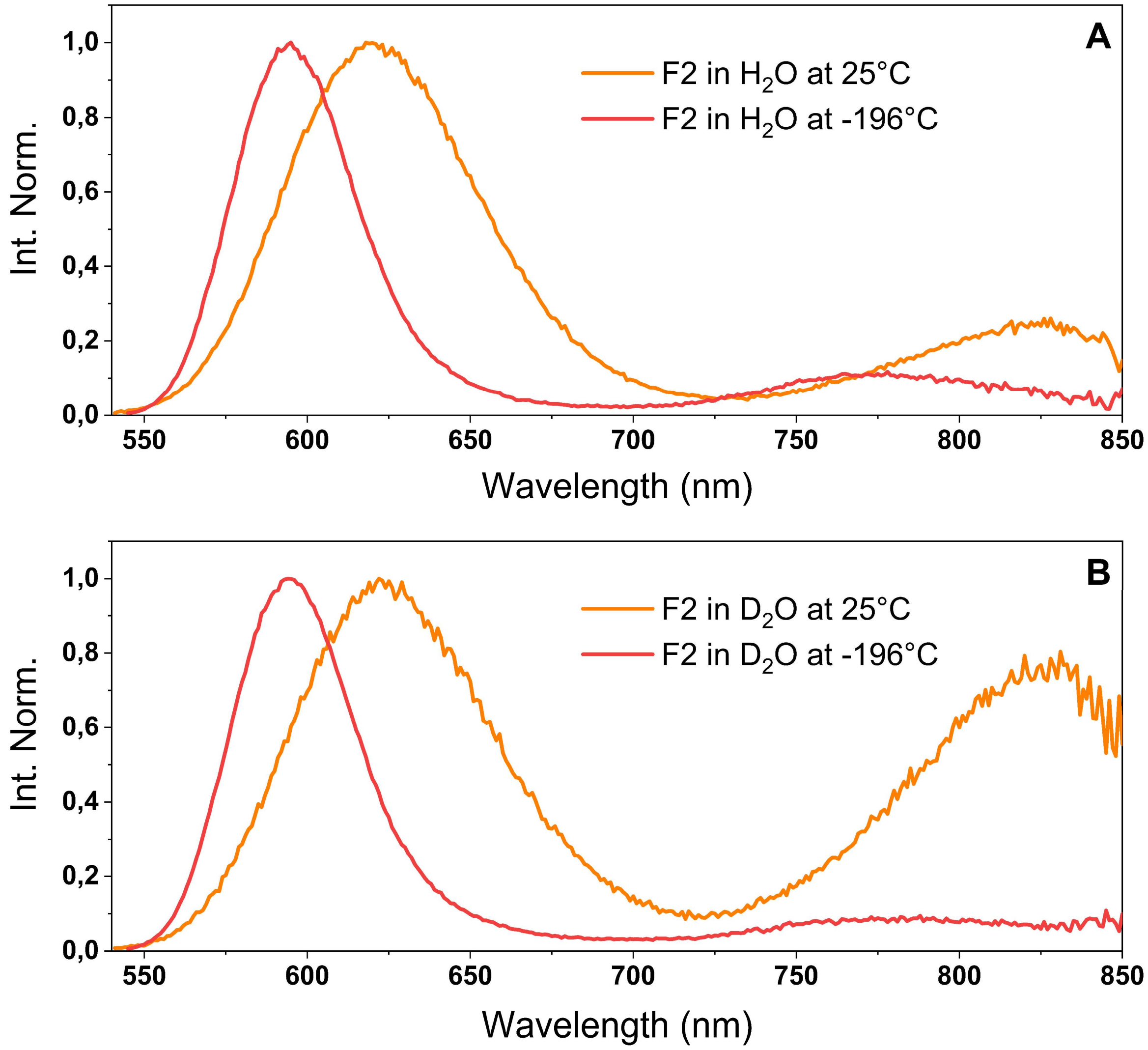

Figure S9. Normalized emission spectra of F2 in **(A)** 10 mM NH_4OAc aqueous solution and (**B)** 10 mM NH_4OAc deuterated solution at 25 °C (orange) and -196 °C (red). The emission spectra were recorded by exciting at 531 nm.

5. Stability at room temperature

The chemical stability of F1 and F2 was evaluated at room temperature by monitoring their optical properties over time. The samples were measured in a 1 cm quartz cuvette. Measurements were carried out in 10 mM NH_4OAc aqueous solution. Specifically, stability was assessed by recording the time-dependent evolution of the UV-visible absorption spectra and the excitation and emission spectra monitored at different emission and excitation wavelengths, respectively. The absorption spectra reported in Figures S10A, C and S11A, C were measured using a Lambda 800 UV/Vis spectrophotometer from Perkin Elmer, while the excitation and emission spectra in Figures S10B, D and S11B, D were recorded with a QuantaMaster 400 Fluorometer (Horiba) equipped with Xenon arc lamp. The spectra were then corrected for the wavelength dependency of the detector and for the wavelength-dependent intensity difference of the excitation lamp. The emission and excitation wavelengths employed for the measurements can be found in the legends of the corresponding spectra. The

main differences between the stability tests shown in Figure S10 and Figure S11 (panels A and B *vs* panels C and D) are related to the experimental conditions. In panels A and B, the cuvettes were not sealed with parafilm, and the samples were monitored on day 0 without consistently keeping them in the dark. The absorption spectra were recorded at 0, 60, 180, 240, and 300 minutes.

In contrast, for panels C and D, the cuvettes were sealed with parafilm to prevent solvent evaporation and minimize gas exchange with the environment. Additionally, particular care was taken to keep the samples protected from light throughout the entire experiment.

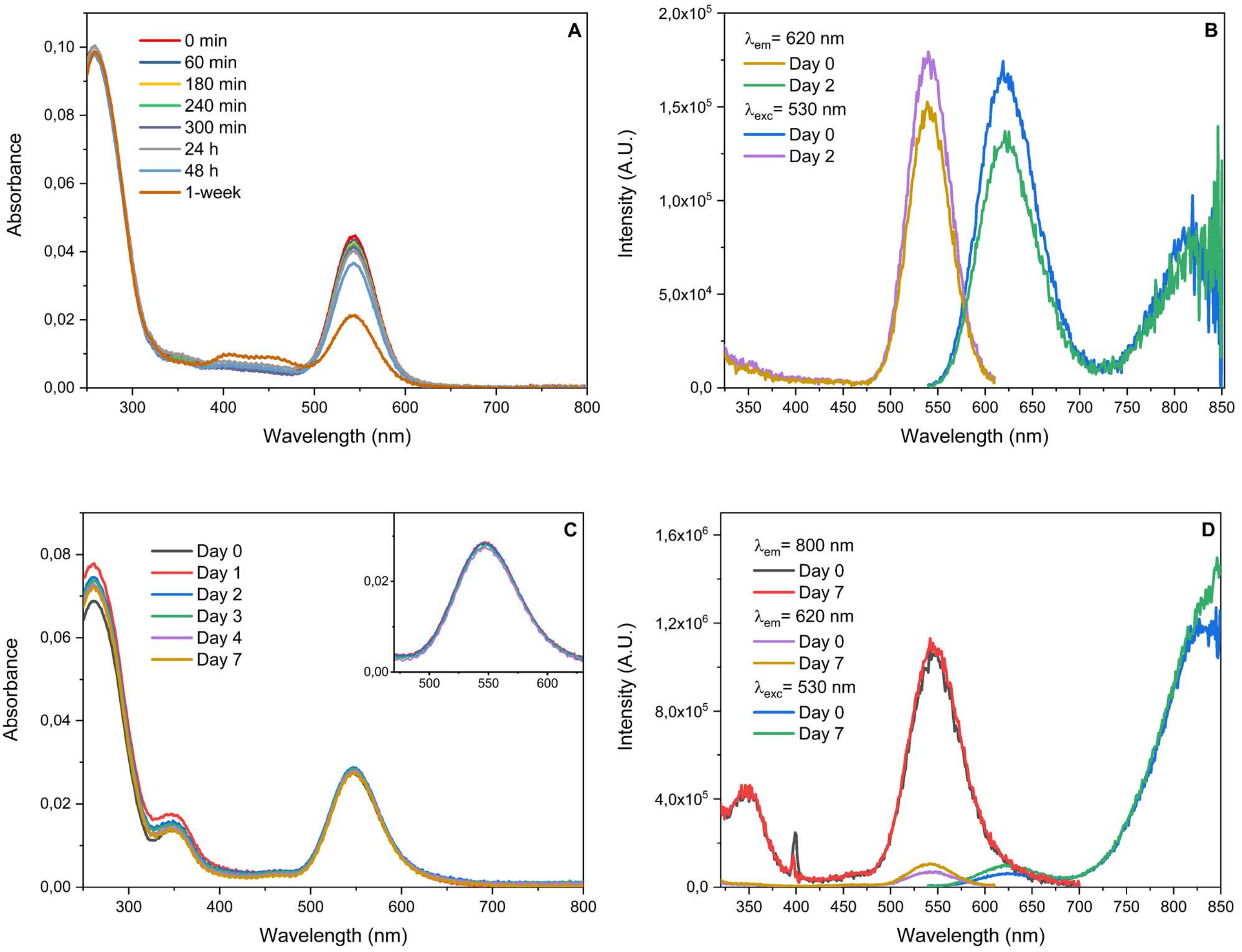

Figure S10. Stability of F1 at room temperature under different experimental conditions. **(A)** Time-dependent UV-visible absorption spectra (0 min to 1 week, during day 0 spectra were acquired at 0, 60, 180, 240, and 300 min) and **(B)** Time-dependent excitation/emission spectra (0 min to 48 h) acquired without sealing the cuvettes with parafilm and without strictly protecting the samples from light (day 0: 0, 60, 180, 240, 300 min); **(C)** UV-vis absorption and **(D)** excitation/emission spectra recorded under the same conditions, but with cuvettes sealed with parafilm to prevent solvent evaporation and gas exchange, and carefully protecting the samples from light exposure.

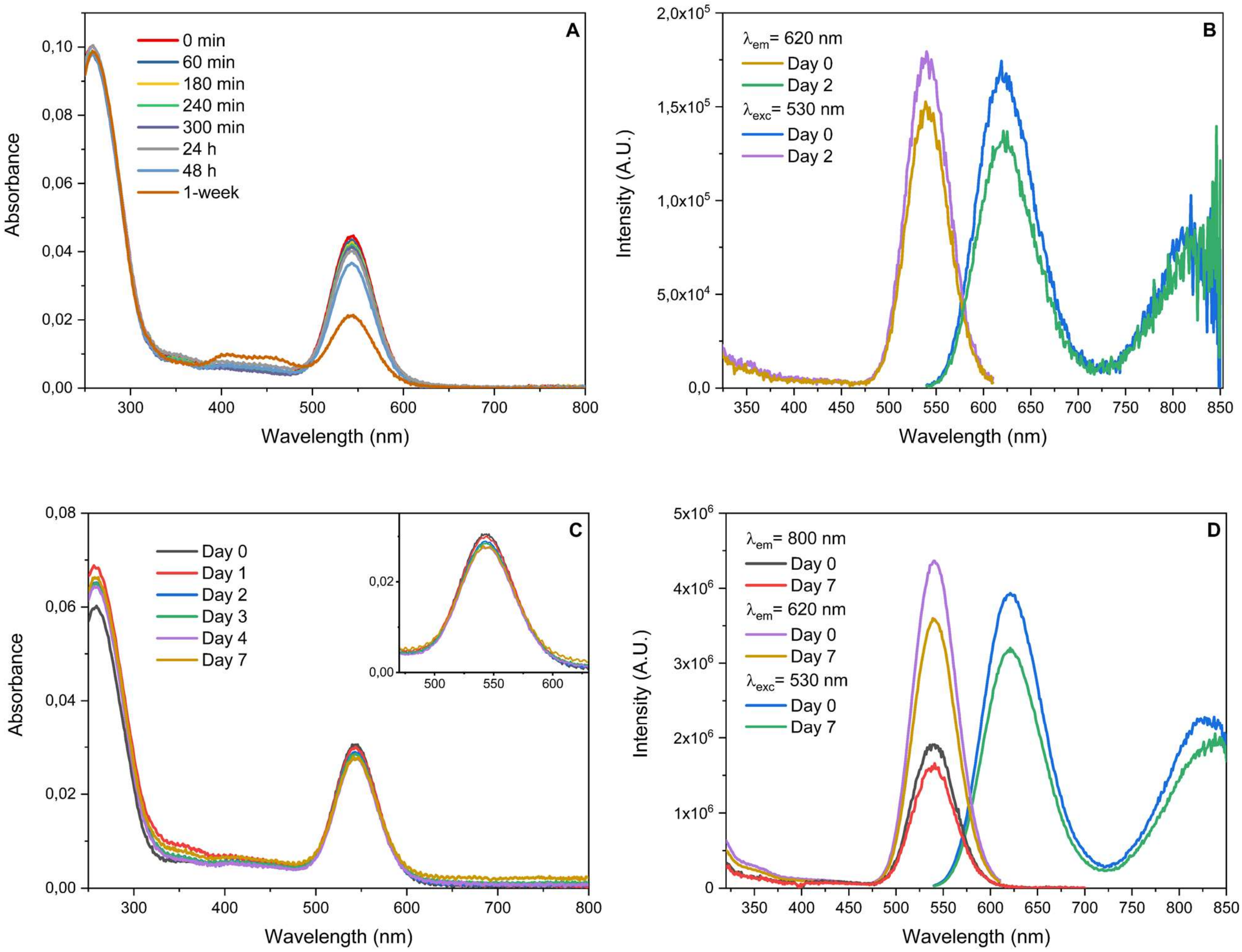

Figure S11. Stability of F2 at room temperature under different experimental conditions. **(A)** Time-dependent UV-visible absorption spectra (0 min to 1 week, during day 0 spectra were measured at 0, 60, 180, 240, and 300 min) and **(B)** Time-dependent excitation/emission spectra (0 min to 48 h) acquired without sealing the cuvettes with parafilm and without strictly protecting the samples from light (day 0: 0, 60, 180, 240, 300 min); **(C)** UV-vis absorption and **(D)** excitation/emission spectra recorded under the same conditions, but with cuvettes sealed with parafilm to prevent solvent evaporation and gas exchange, and carefully protecting the samples from light exposure.

6. Electrospray-ionization mass spectrometry (ESI-MS)

The ESI-MS data were acquired with a Xevo G2-XS QTof (Waters Corporation), using negative ion mode with a 1.5 kV capillary voltage, 30 V cone voltage, 80 V source offset and collision mode set to off. Spectra were collected from m/z 750 to 4000, with a scan time of 1 s. The source temperature was 100 °C with a cone gas flow of 50 L/h, and the desolvation temperature and gas flow were 350 °C and 800 L/h, respectively. The QTof was calibrated using ESI-L Low Tune Mix (Agilent Technologies), which contained compounds for negative mode in the mass range of m/z 1034 to 2834. The sample was injected using an Acquity I-Class Plus system (Waters) with a flow-through needle autosampler, with a flow of 0.05 mL/min 50 mM NH_4OAc at pH 7 MeOH (80:20) and using 3 µL injection volume. In Figure S15B, a comparison between the ionization conditions used in a previous study by *Rück et al.*[1] is presented. Specifically, ESI-MS measurements were performed using the same spectrometer in negative ion mode with 30 V cone voltage and no collision energy as in this work, but with a 2 kV capillary voltage. Spectra were collected over an m/z range of 1000–4000 with a scan time of 1 s. The source temperature was 80 °C, with a cone gas flow of 45 L/h, while the desolvation temperature and gas flow were 150 °C and 450 L/h, respectively. The QTof was calibrated using the same standard described above, and the same injection system was employed. The flow rate was set to 0.1 mL/min

using the same solvent, and the injection volume was chosen to be 5 μL. In both cases, the system was operated using UNIFI v.1.9.4 (Waters), and the final spectra were generated by averaging multiple spectra surrounding the apex of the observed peak. All the recorded data were analyzed with the open-source software enviPat (https://www.envipat.eawag.ch/index.php), together with a self-written MATLAB script. The MATLAB script was used to identify the maximum of each isotope peak of them/z peaks in order to fit them (Figures 3B, 3C, S13, and S14) with single Gaussian functions. Adjacent peaks of the isotope pattern are spaced by 1/z, which defines the charge state **-eZ** of a *m/z* peak. The total charge of the DNA-AgNC corresponding to a certain *m/z* peak is equal to the number of silver cations, $\mathbf{eN_+}$, minus the number of protons removed from the DNA, $\mathbf{en_{pr}}$, to reach the total charge of **-eZ** observed experimentally: $\mathbf{-eZ = eN_+ - en_{pr}}$; where **e** is the elementary charge. Since $\mathbf{n_{pr}}$ protons are removed from the DNA-AgNC, the experimentally measured total mass **m** (in amu) is given by $\mathbf{m = m_{DNA} \cdot n_s + m_{Ag} \cdot (N_+ + N_0) - n_{pr}}$; where $\mathbf{N_+}$ is the number of silver cations, $\mathbf{N_0}$ is the number of reduced silver atoms, $\mathbf{m_{DNA}}$ is the DNA mass, $\mathbf{n_s}$ is the number of DNA strands, and $\mathbf{m_{Ag}}$ is the silver atom mass. By varying the $\mathbf{N_+}$ values, and thus $\mathbf{n_{pr}}$, it is possible to determine the nanocluster charge (and the number of reduced silvers) that best matches the observed isotope pattern.

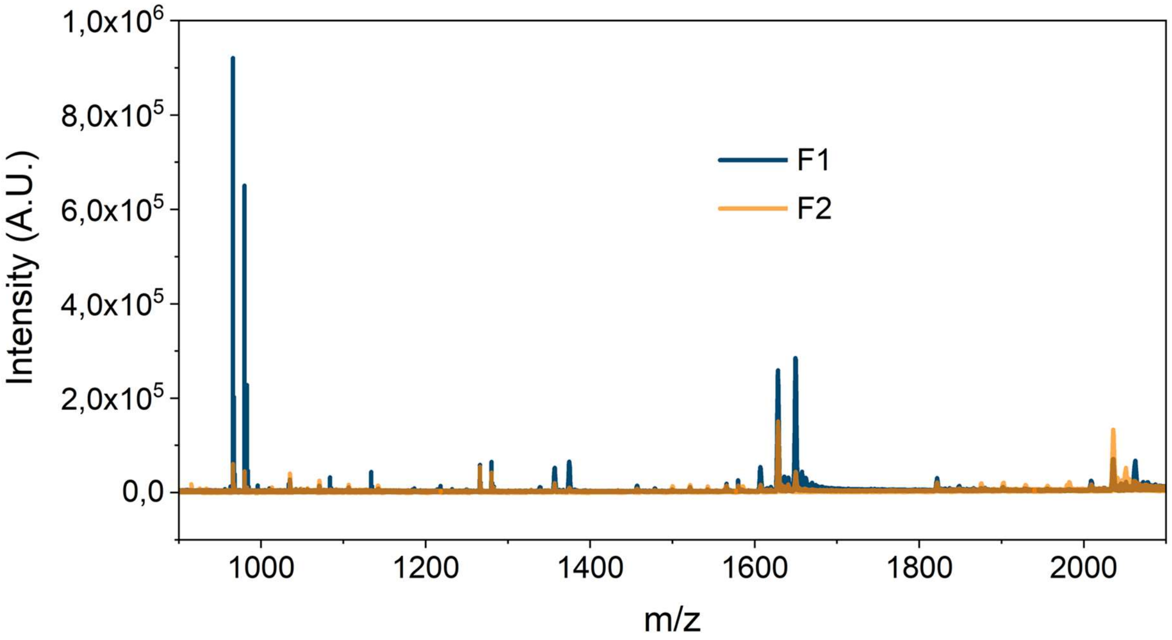

Figure S12. Mass spectra of F1 (blue) and F2 (orange).

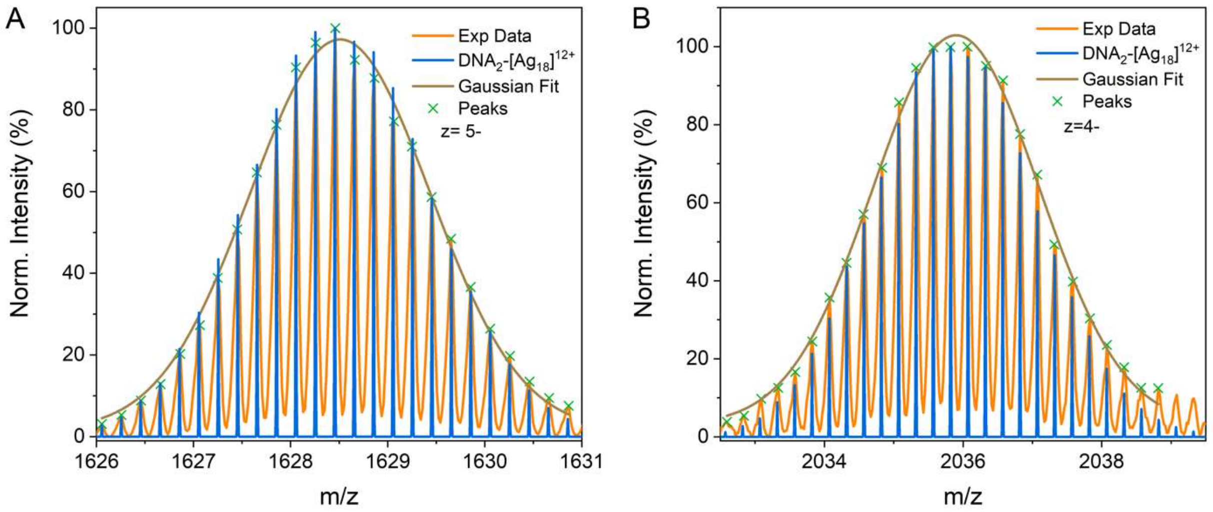

Figure S13. Zoomed-in view of the mass spectrum reported in Figure S12 for F1. The experimental isotopic distribution (orange) is reported with the corresponding Gaussian fit (ochre) and the theoretical isotopic distribution (blue) for $DNA_2[Ag_{18}]^{12+}$ corresponding to **(A)** z=5$^-$, and **(B)** z=4$^-$. The experimental average masses are *m/z* 1628.511 ± 0.011 and 2035.899 ± 0.012, respectively. See Table S3 for the calculations.

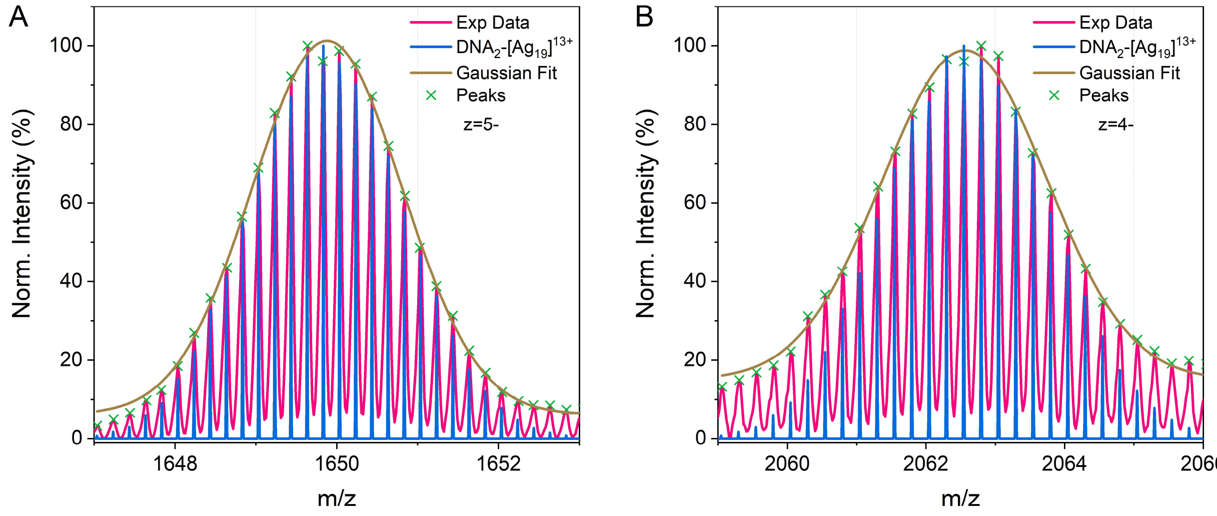

Figure S14. Zoomed-in view of the mass spectrum reported in Figure S12 for F1. The experimental isotopic distribution (pink) is reported with the corresponding Gaussian fit (ochre) and the theoretical isotopic distribution (blue) for $DNA_2[Ag_{19}]^{13+}$ corresponding to **(A)** z=5$^-$, and **(B)** z=4$^-$. The experimental average masses are *m/z* 1649.881 ± 0.009 and 2062.561 ± 0.015, respectively. See Table S3 below, for the calculations.

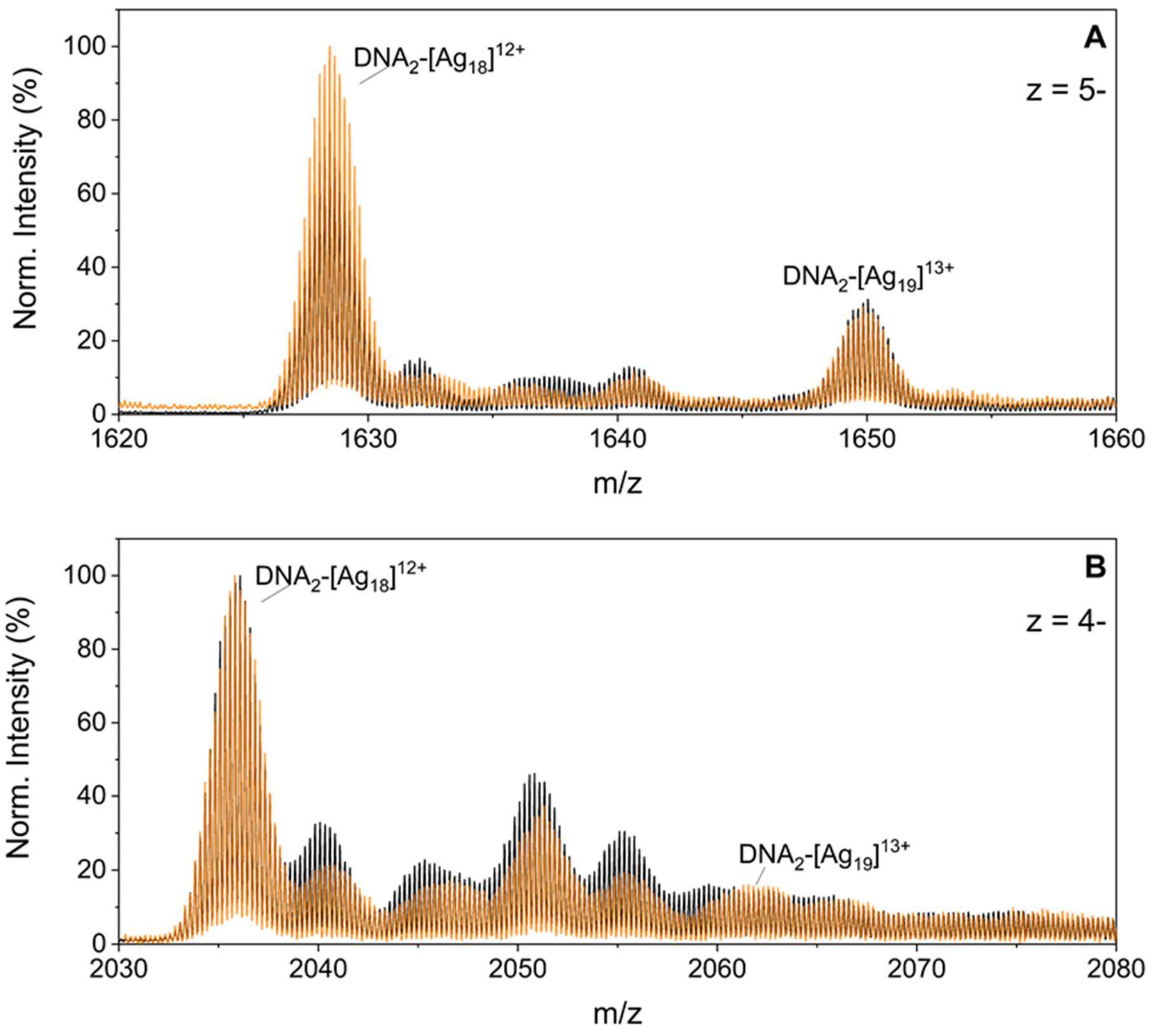

Figure S15. Comparison of the ionization conditions used by Rück *et al.*[1] (black) with those employed in this study (orange). Zoomed-in views of **(A)** $z=5^-$ and **(B)** $z=4^-$ regions of the mass spectra recorded for F2.

Table S3. Center of Gaussian fits, x_0, for the experimentally measured mass spectra (x_0^{exp}) shown in Figures 3B, 3C, S13-14, and the corresponding theoretical mass distributions (x_0^{th}). The last column corresponds to the absolute error calculated as $x_0^{exp}- x_0^{th}$.

Molecular formula	z	Chemical Formula	Molecular weight*	x_0^{th}	x_0^{exp}	Error
DNA_2-$[Ag_{18}]^{12+}$	4-	$C_{196}H_{244}N_{86}O_{116}P_{18}[Ag_{18}]^{12+}$	8159,74 g/mol	2035,868 ± 0,005	2035,899 ± 0,012	+ 0,031
	5-	$C_{196}H_{244}N_{86}O_{116}P_{18}[Ag_{18}]^{12+}$	8159,74 g/mol	1628,493 ± 0,005	1628,511 ± 0,011	+ 0,006
DNA_2-$[Ag_{19}]^{13+}$	4-	$C_{196}H_{244}N_{86}O_{116}P_{18}[Ag_{19}]^{13+}$	8267,61 g/mol	2062,584 ± 0,005	2062,561 ± 0,015	- 0,023
	5-	$C_{196}H_{244}N_{86}O_{116}P_{18}[Ag_{19}]^{13+}$	8267,61 g/mol	1649,881 ± 0,009	1649,872 ± 0,064	- 0,009

* As determined by ChemDraw Pro 8.0.

7. Crystallization, single-crystal X-ray diffraction measurements and data analysis

7.1 Crystallization

The hanging-drop vapor diffusion method was used to obtain crystals at room temperature. 0,5-1 μL of DNA-AgNC solution, with [DNA-AgNC]≈100-200 μM, were mixed with 0,5-1 μL of crystallization buffer and equilibrated against 250 μL of a reservoir solution, either 40% 2-methyl-2,4-pentanediol (MPD) or 40% poly-ethylene glycol (PEG) 3350. The crystallization buffer contains either 10% MPD or 10% PEG 3350, a nitrate salt (Li^+, Na^+, K^+, NH_4^+, Mg^{2+}, Ca^{2+} or Sr^{2+}) with different concentrations (10, 100, 200, 300, 400 and 500 mM), 10 mM spermine and 50 mM 3-(N-morpholino)propanesulfonic acid (MOPS) at pH 7. Crystals were obtained between 1 and 2 weeks after starting the crystallization. Examples are reported in Figures S16 and S17.

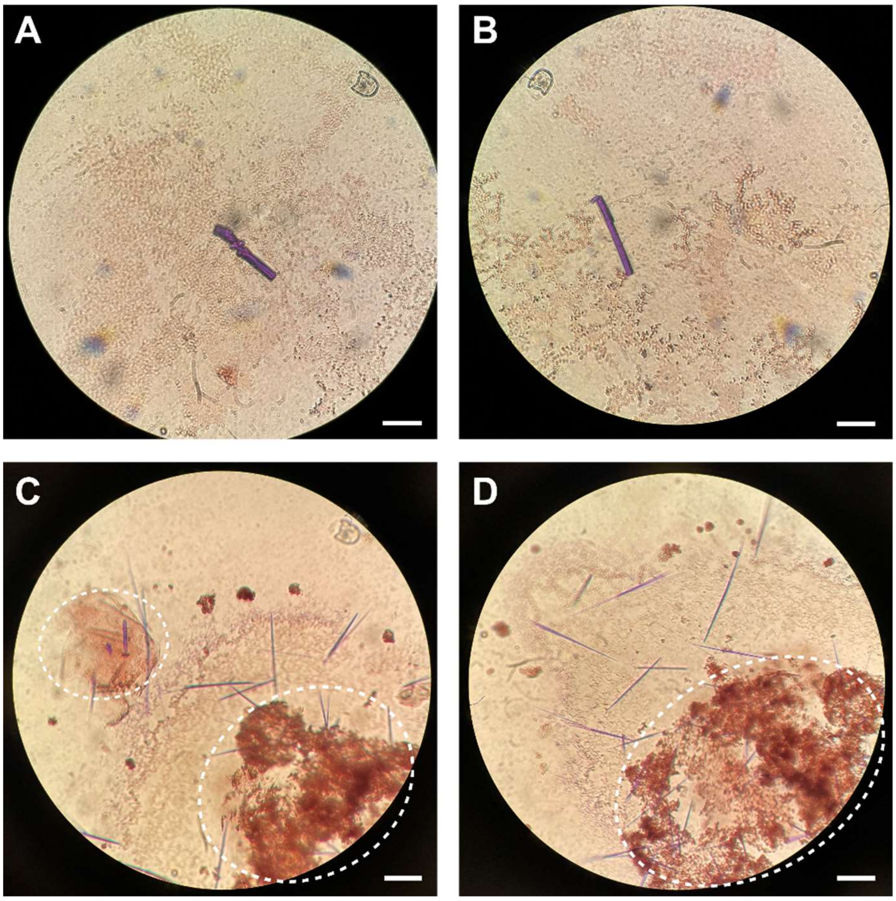

Figure S16. Examples of F2 crystals **(A, B)** with limited light exposure (within the first minutes) and **(C, D)** after prolonged light exposure (1-5 min) on the microscope. All images were acquired using an iPhone 12 mini camera. Panels (C) and (D) correspond to different crystallization wells than (A) and (B). All crystals were grown in 10% MPD, 10 mM spermine, 50 mM MOPS (pH 7), and 100 mM NH_4NO_3. White dashed-line circles highlight regions of melted crystals. The scale bar is 0.1 mm in all images.

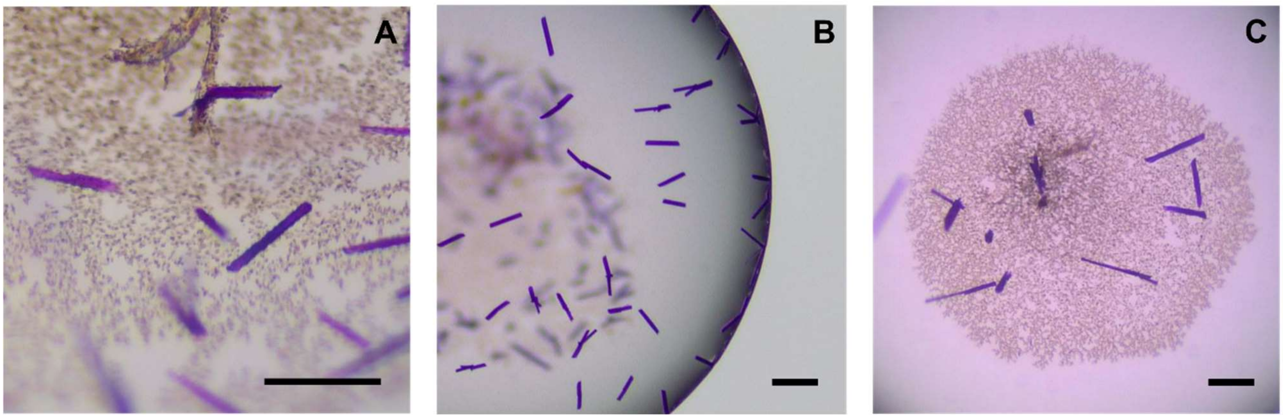

Figure S17. Examples of F1 crystals grown in: **(A)** 10% PEG 3350, 10 mM spermine, 50 mM MOPS (pH 7) and 100 mM $NaNO_3$; **(B)** 10% PEG 3350, 10 mM spermine, 50 mM MOPS (pH 7) and 200 mM NH_4NO_3; and **(C)** 10% PEG 3350, 10 mM spermine, 50 mM MOPS (pH 7) and 200 mM $Sr(NO_3)_2$. The scale bar is 0.1 mm in all images.

7.2 Single-crystal X-ray diffraction measurements and data analysis

To determine the structure of the $DNA_2[Ag_{17+2}]^{13+}$, we collected datasets using the BioMAX beamline at MAX IV (Lund, Sweden). The diffraction patterns were obtained using 0.1° oscillation steps with an exposure time of 0.01 s. A total of 3600 frames were recorded. All datasets were processed with the program *XDS*.[5] The reflection data were converted by *Reflection file editor* of the *Phenix suite*.[6] The initial phase and partial silver cluster structure were determined by the standard direct method phasing protocol in *SIR2019*.[7] Using the partial silver cluster as a reference for the heavy atom sites, the program *AutoSol* in the *Phenix suite*[6] was used to estimate the complete silver cluster structure and to observe the electron density map corresponding to the DNA bases. The crystal structure was constructed by using the program *Coot*.[8, 9] The atomic parameters were refined using the program *phenix.refine* in the *Phenix suite*.[6] Table S4 lists the crystallization conditions that yielded the crystal used for data collection, whereas Table S5 summarizes the crystal parameters, as well as the data collection and structure refinement statistics. All structural figures were generated by *PyMOL.*[10]

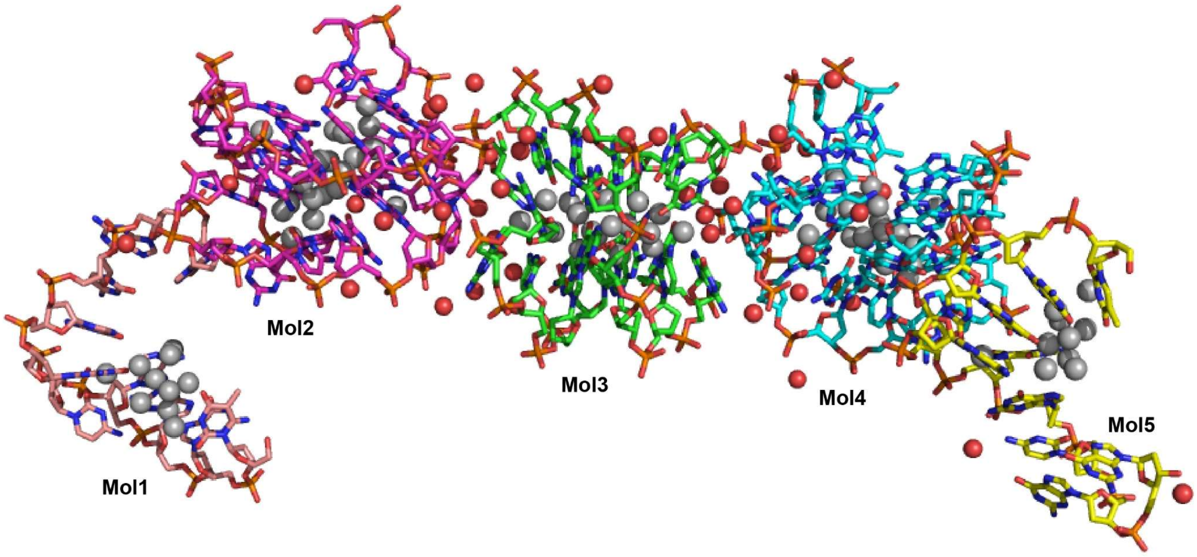

Figure S18. Asymmetric unit of the measured F1 crystal composed of three DNA-AgNCs (Mol2, Mol3, and Mol4 – where Mol stands for molecule) and two half DNA-AgNCs (Mol1 and Mol5). The gray spheres represent the silver atoms, while the red spheres are the oxygen atoms of water molecules present in the unit.

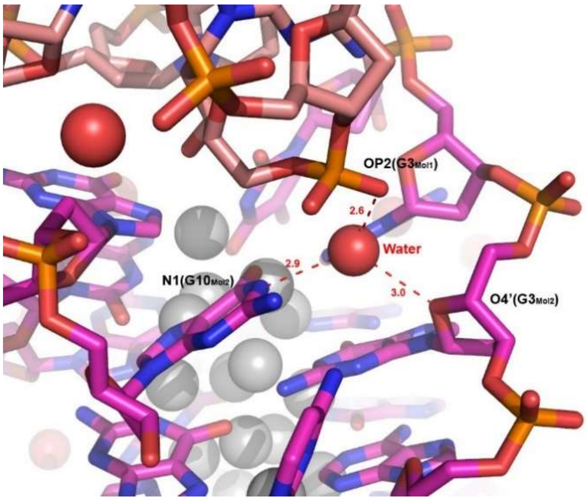

Figure S19. Mol1 and Mol2 interact *via* three hydrogen bonds, N1($H_{G10, Mol2}$)-O_{water}, O(H_{water})-O($P2_{G3, Mol1}$), and O(H_{water})-O4'$_{G3, Mol2}$.

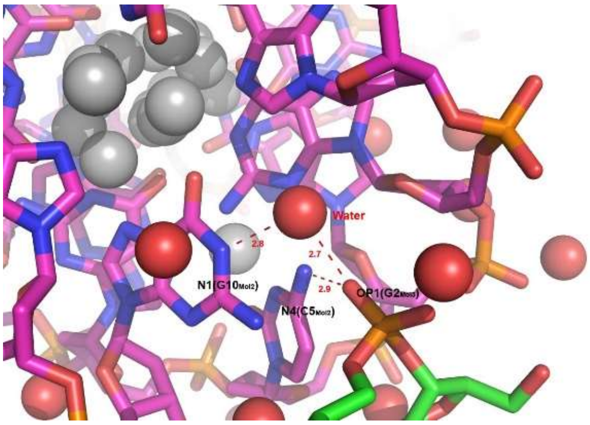

Figure S20. Between Mol2 and Mol3, the crystal packing is enhanced by three hydrogen bonds, N1($H_{G1, Mol2}$)-O_{water}, O(H_{water})-O($P1_{G2, Mol3}$), and N4($H_{C5, Mol2}$)-O($P1_{G2, Mol3}$).

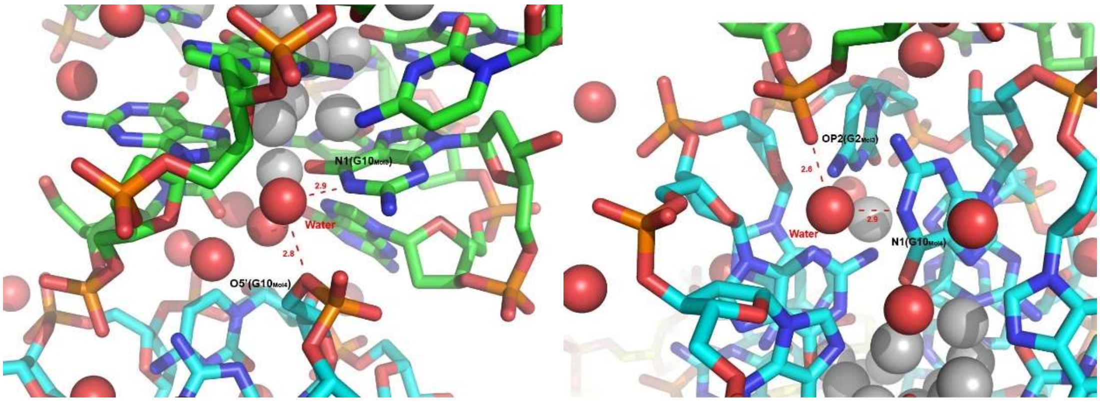

Figure S21. Between Mol3 and Mol4, two water molecules allow for the crystal packing interactions *via* four hydrogen bonds, N1($H_{G10, Mol3}$)-O_{water}, O(H_{water})-O5'$_{G10, Mol4}$, O(H_{water})-O($P2_{G2, Mol3}$), and N1($H_{G10, Mol4}$)-O_{water}.

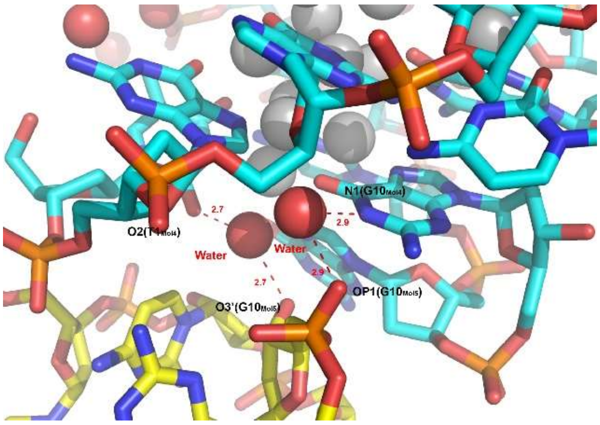

Figure S22. Between Mol4 and Mol5, two water-mediated interactions are observed, N1($H_{G10,\ Mol4}$)-O_{water}, OH_{water}-$OP1_{G10,\ Mol5}$, OH_{water}-$O2_{T1,\ Mol4}$, and OH_{water}-$O3'_{G10,\ Mol5}$.

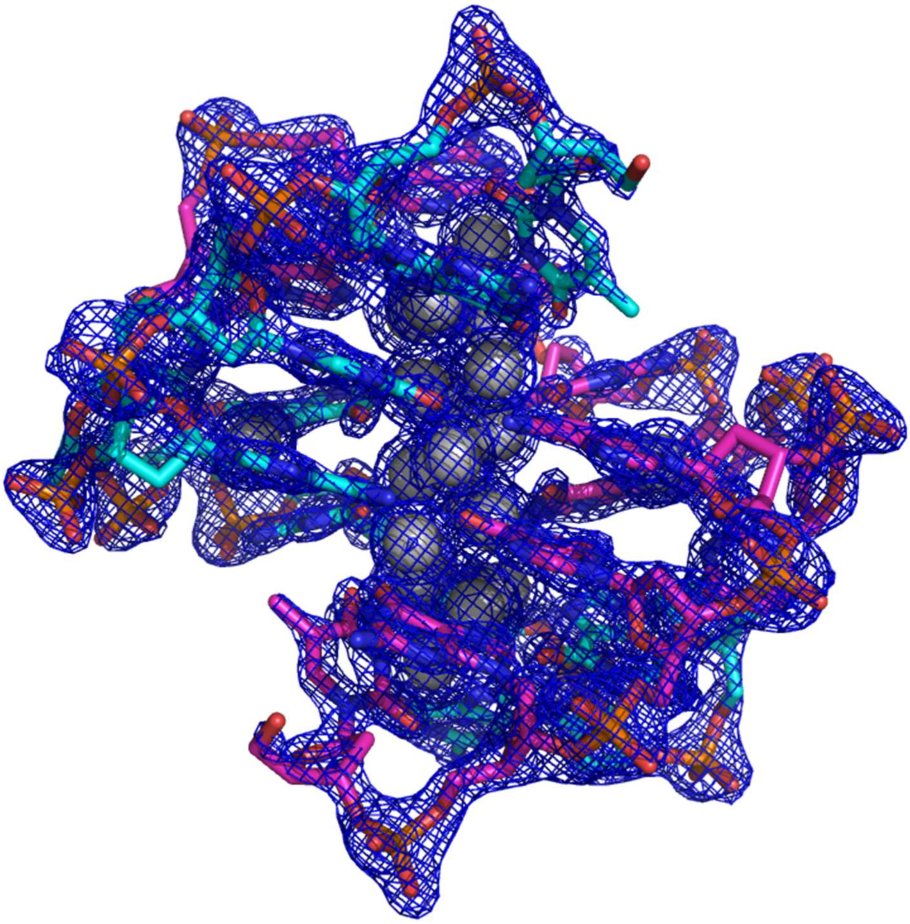

Figure S23. 2|Fo|-|Fc| map of Mol3 drawn at 1σ contour level.

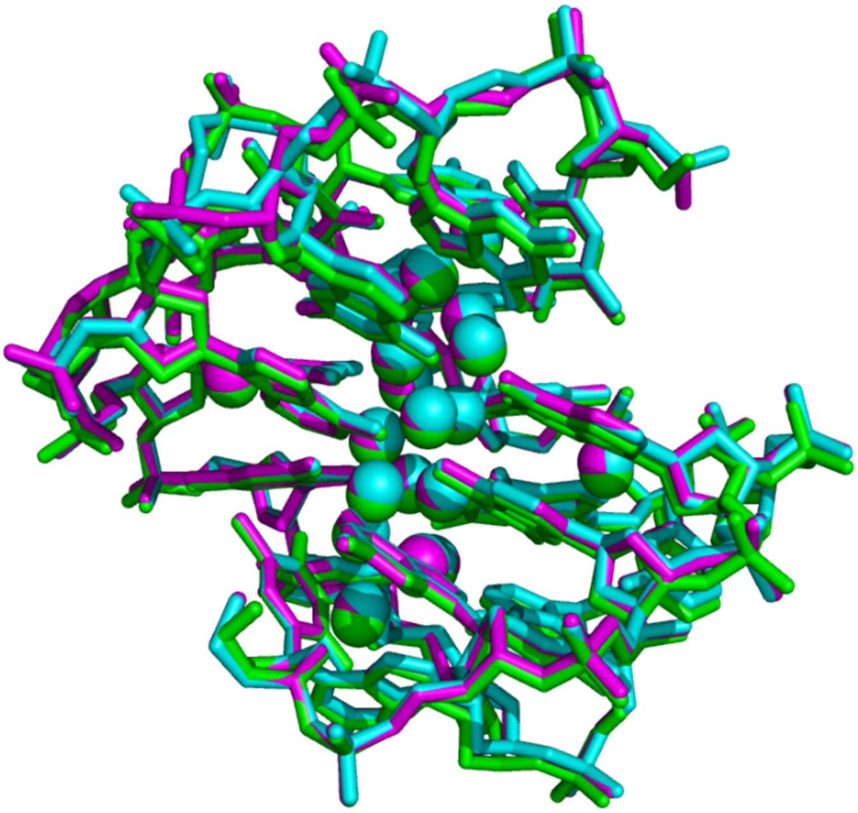

Figure S24. Superposition of the structures of Mol2 (magenta), Mol3 (green), and Mol4 (blue). The RMSDs are 0.083 (Mol2 *vs* Mol3), 0.085 (Mol3 *vs* Mol4), and 0.015 (Mol2 *vs* Mol4), implying these structures are almost identical.

Table S4. Crystallization conditions for X-ray diffraction data collection of the F1 crystal.

DNA-AgNC solution (1 µL)	
[DNA-AgNC]	≈100-200 µM
Crystallization solution (1µL)	
MOPS (pH = 7.0)	50 mM
Spermine	10 mM
Ammonium nitrate	300 mM
PEG 3350	10%
Reservoir solution (250 µL)	
PEG 3350	40%
Crystal	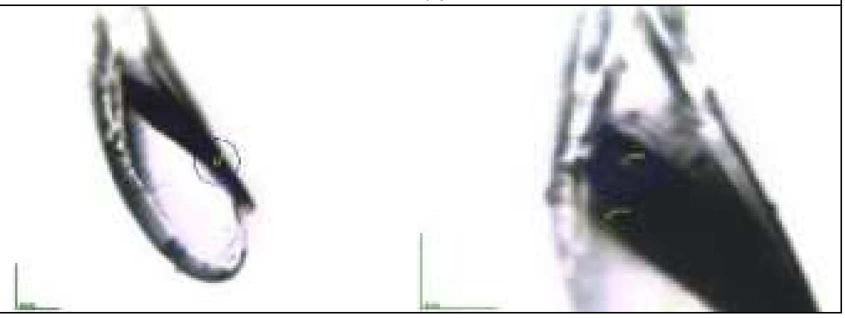

Table S5. Crystal data, statistics of data collection and structure refinement for F1.

PDB-ID	23LP (pdb_000023lp)
Crystal data	
Space group	$C2$
Unit cell (Å, °)	a = 30,35 ; b = 53,92 ; c = 138,14 ; β = 89,97
Z [a]	4
Data collection	
Beamline	BioMAX of MAX IV
Wavelength (Å)	0,8
Resolution (Å)	26,46-1,7
of the outer shell (Å)	1,76-1,70
Unique reflections	47494
Completeness (%)	98,4
in the outer shell (%)	97,8
R_{anom} [b] (%)	6,0
in the outer shell (%)	22,8
Redundancy	3,53
in the outer shell	3,50
Structure refinement	
Resolution range (Å)	26,46-1,7
Used reflections	47480
R-factor [c] (%)	18,1
R_{free} [d] (%)	19,3
Number of DNA atoms	1664
Number of Ag	77
Number of water	48
R.m.s.d. bond length (Å)	0,010
R.m.s.d. bond angles (°)	1,3

[a] Number of DNA-AgNC in the asymmetric unit.
[b] $R_{anom} = 100 \times \Sigma_{hklj}|I_{hklj}(+)–I_{hklj}(–)| / \Sigma_{hklj}[I_{hklj}(+) + I_{hklj}(–)]$.
[c] R-factor = $100 \times \Sigma||F_o| – |F_c|| / \Sigma|F_o|$, where $|F_o|$ and $|F_c|$ are optimally scaled observed and calculated structure factor amplitudes, respectively.
[d] Calculated using a random set containing 10% of observations.

7.2 Photophysical properties of F1 crystals

Emission spectra were recorded on our home-built confocal microscope (Olympus IX71) by placing the lid of the crystallization plate containing the crystals on top of a glass coverslip.[2] A continuum white-light laser (SuperK EXTREME EXB-6, NKT Photonics) was used as an excitation source delivering a wavelength of 520 nm with a 12,98 MHz repetition rate by sending the continuum output through an acousto-optic tunable filter (SuperK SELECT, NKT Photonics). The output of the laser was expanded and collimated by a lens system and cleaned up by a band-pass filter (FF01-520/5-25, Semrock) before it was reflected by a 30:70 beam splitter (XF122, Omega Optical) and sent through an air objective (CPlanFLN 10x NA = 0.3, Olympus). The objective that collected the fluorescence was directed through a 100 µm pinhole and a 532 nm long-pass filter (BLP01-532R-25, Semrock). The fluorescence was then sent through a spectrograph (SP 2356 spectrometer, 300 grooves/mm, Acton Research) onto a nitrogen cooled CCD camera (SPEC-10:100B/LN-eXcelon, Princeton Instruments) to record the spectra. Finally, the emission spectra were wavelength and intensity corrected as reported previously.[3]

The decay curves were recorded on the same experimental setup but in the excitation path an additional band-pass filter (FF01-527/20-25, Semrock) and a long-pass filter (LP02-488RU-25, Semrock) were used. The extraction of macro-time data follows the methodology reported in reference 11.

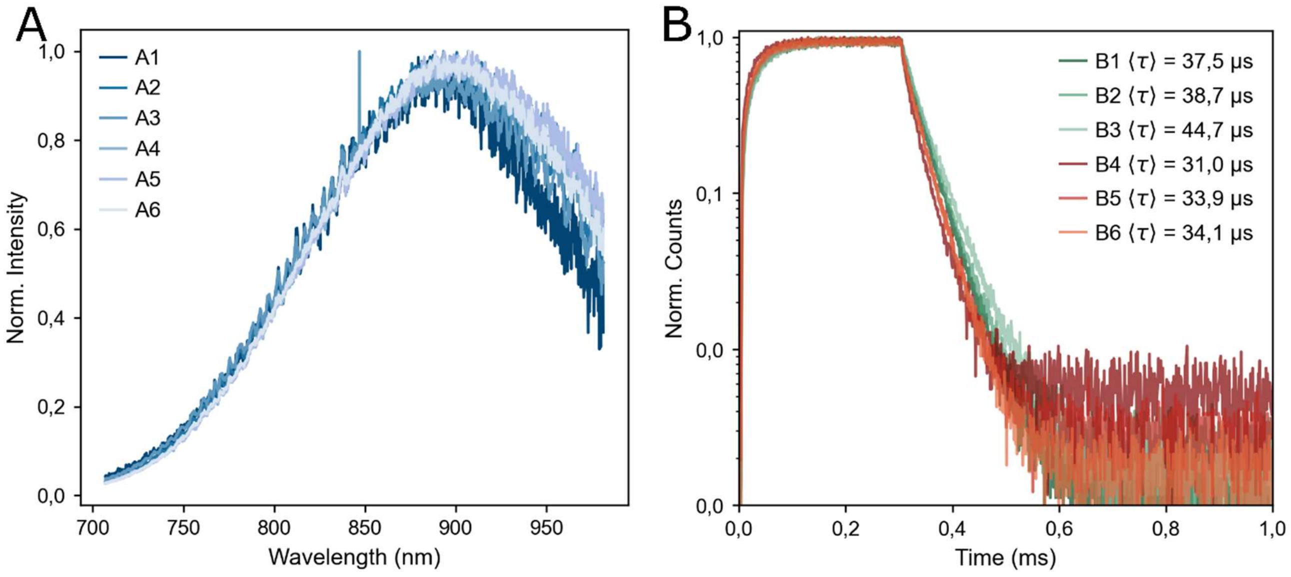

Figure S25. Photophysical data of F1 crystals. **(A)** Steady-state emission spectra of several F1 crystals (A1 to A6) showing peaks centered at ~890 nm, red-shifted with respect to the solution state. The red tails of the spectra were not captured because of limited detector efficiency above 950 nm. Crystal A1 was measured with a 60 s acquisition time, whereas crystals A2-A6 were measured for 30 s. Crystals A1-A5 were grown in 200-300 mM NH_4NO_3 (A1 and A2 in 200 mM NH_4NO_3; A3-A5 in 300 mM NH_4NO_3), 10% PEG 3350, 10 mM spermine and 50 mM MOPS (pH 7); while crystal A6 was grown in 10 mM $Sr(NO_3)_2$, 10% MPD, 10 mM spermine and 50 mM MOPS (pH 7). **(B)** Time-resolved luminescence decays measured with a burst mode approach. The intensity-weighted average decay times $<\tau>_{\mu s}$ reported in the legend were determined by tail-fitting the data with bi-exponential functions. Green curves (B1-B3) correspond to crystals grown in 100 mM NH_4NO_3, 10% PEG 3350, 10 mM spermine and 50 mM MOPS (pH 7), while red curves (B4-B6) correspond to crystals grown in 10 mM $Ca(NO_3)_2$, 10% PEG 3350, 10 mM spermine and 50 mM MOPS (pH 7).

References

1. Rück, V.; Liisberg, M. B.; Mollerup, C. B.; He, Y.; Chen, J.; Cerretani, C.; Vosch, T., A DNA-Stabilized Ag1812+ Cluster with Excitation-Intensity-Dependent Dual Emission. *Angewandte Chemie International Edition* **2023,** *62* (39), e202309760.
2. Liisberg, M. B.; Shakeri Kardar, Z.; Copp, S. M.; Cerretani, C.; Vosch, T., Single-Molecule Detection of DNA-Stabilized Silver Nanoclusters Emitting at the NIR I/II Border. *The Journal of Physical Chemistry Letters* **2021,** *12* (4), 1150-1154.
3. Brouwer, A. M., Standards for photoluminescence quantum yield measurements in solution (IUPAC Technical Report). *Pure and Applied Chemistry* **2011,** *83* (12), 2213-2228.
4. Lakowicz, J. R., *Principles of fluorescence spectroscopy*. 2006; p 1-954.
5. Kabsch, W., XDS. *Acta Crystallographica Section D* **2010,** *66* (2), 125-132.
6. Liebschner, D.; Afonine, P. V.; Baker, M. L.; Bunkoczi, G.; Chen, V. B.; Croll, T. I.; Hintze, B.; Hung, L.-W.; Jain, S.; McCoy, A. J.; Moriarty, N. W.; Oeffner, R. D.; Poon, B. K.; Prisant, M. G.; Read, R. J.; Richardson, J. S.; Richardson, D. C.; Sammito, M. D.; Sobolev, O. V.; Stockwell, D. H.; Terwilliger, T. C.; Urzhumtsev, A. G.; Videau, L. L.; Williams, C. J.; Adams, P. D., Macromolecular structure determination using X-rays, neutrons and electrons: recent developments in Phenix. *Acta Crystallographica Section D* **2019,** *75* (10), 861-877.
7. Burla, M. C.; Caliandro, R.; Carrozzini, B.; Cascarano, G. L.; Cuocci, C.; Giacovazzo, C.; Mallamo, M.; Mazzone, A.; Polidori, G., Crystal structure determination and refinement via SIR2014. *Journal of Applied Crystallography* **2015,** *48* (1), 306-309.
8. Emsley, P.; Cowtan, K., Coot: model-building tools for molecular graphics. *Acta Crystallographica Section D* **2004,** *60* (12 Part 1), 2126-2132.
9. Emsley, P.; Lohkamp, B.; Scott, W. G.; Cowtan, K., Features and development of Coot. *Acta Crystallographica Section D* **2010,** *66* (4), 486-501.
10. DeLano, W. L., The PyMOL Molecular Graphics System. DeLano Scientific LLC, Palo Alto, CA, USA. **2008**.
